# Metformin alleviates human cellular aging by upregulating the endoplasmic reticulum glutathione peroxidase 7

**DOI:** 10.1111/acel.12765

**Published:** 2018-04-16

**Authors:** Jingqi Fang, Jiping Yang, Xun Wu, Gangming Zhang, Tao Li, Xi'e Wang, Hong Zhang, Chih‐chen Wang, Guang‐Hui Liu, Lei Wang

**Affiliations:** ^1^ National Laboratory of Biomacromolecules CAS Center for Excellence in Biomacromolecules Institute of Biophysics Chinese Academy of Sciences Beijing China; ^2^ College of Life Sciences University of Chinese Academy of Sciences Beijing China; ^3^ National Clinical Research Center for Geriatric Disorders Xuanwu Hospital of Capital Medical University Beijing China

**Keywords:** aging, glutathione peroxidase 7, metformin, nuclear factor erythroid 2‐related factor 2, oxidative stress, senescence

## Abstract

Metformin, an FDA‐approved antidiabetic drug, has been shown to elongate lifespan in animal models. Nevertheless, the effects of metformin on human cells remain unclear. Here, we show that low‐dose metformin treatment extends the lifespan of human diploid fibroblasts and mesenchymal stem cells. We report that a low dose of metformin upregulates the endoplasmic reticulum‐localized glutathione peroxidase 7 (GPx7). GP×7 expression levels are decreased in senescent human cells, and GPx7 depletion results in premature cellular senescence. We also indicate that metformin increases the nuclear accumulation of nuclear factor erythroid 2‐related factor 2 (Nrf2), which binds to the antioxidant response elements in the *GPX7* gene promoter to induce its expression. Moreover, the metformin‐Nrf2‐GPx7 pathway delays aging in worms. Our study provides mechanistic insights into the beneficial effects of metformin on human cellular aging and highlights the importance of the Nrf2‐GPx7 pathway in pro‐longevity signaling.

## 1 INTRODUCTION

Aging is broadly defined as the time‐dependent functional decline of a living organism. Recently, hallmarks of aging, including cellular senescence and stem cell exhaustion, were identified (Lopez‐Otin, Blasco, Partridge, Serrano, & Kroemer, 2013), guiding the research on aging and aging intervention strategies. Furthermore, the first clinical trial, Target Aging with Metformin (TAME), was approved to create a paradigm for evaluating pharmacologic approaches to delay aging (Barzilai, Crandall, Kritchevsky, & Espeland, 2016). The biguanide drug metformin, which is an FDA‐approved first‐line drug for treating type 2 diabetes mellitus, has been used clinically for over 60 years for its effectiveness, safety, and low cost (Bailey & Day, 2004); now, metformin is being considered as a promising geroprotector candidate.

The data indicating that metformin can be used as a potential geroprotector show that it is effective in alleviating various age‐associated disorders, including cardiovascular disease, cancer, and cognitive decline, and decreasing the number of deaths of elderly diabetic patients (Bannister et al., 2014). In addition, the effects of metformin on elongating lifespan have been demonstrated in animal models, including worms (Cabreiro et al., 2013; De Haes et al., 2014; Onken & Driscoll, 2010; Wu et al., 2016), mice (Martin‐Montalvo et al., 2013), and rats (Smith et al., 2010). The current consensus is that metformin targets multiple cellular signaling pathways closely associated with the development of aging, such as inflammation, cellular survival, stress defense, autophagy, and protein synthesis (Barzilai et al., 2016). One well‐accepted mechanism of metformin‐mediated lifespan extension is its ability to mimic the effects of dietary restriction through stimulating adenosine monophosphate‐activated protein kinase (AMPK), the principal energy sensor in cells, to reduce energy‐consuming processes (Martin‐Montalvo et al., 2013; Onken & Driscoll, 2010; Zhou et al., 2001). Nevertheless, whether metformin can suppress human cellular aging and the mechanisms underlying its probable geroprotective effects in humans remain unclear.

Living organisms are exposed to reactive oxidants from their internal metabolism and the environment. Although reactive oxygen species (ROS) at normal levels function as specific regulators of multiple signaling pathways (Finkel, 2011), the excessive accumulation of ROS may cause biomacromolecule damage and cell toxicity (Ma, 2013). Hence, cells in many different species have developed a robust antioxidant system to maintain redox homeostasis and to relieve oxidative stress. Nuclear factor erythroid 2‐related factor 2 (Nrf2) has been identified as an evolutionarily conserved transcription factor critical for regulating many antioxidant responses; this action occurs via Nrf2 binding to the antioxidant response elements (AREs) in the promoter regions of target genes (Ma, 2013). Impaired Nrf2‐ARE activity was observed not only in physiological aging (Suh et al., 2004) but also in premature aging disorders (Kubben et al., 2016). In *Caenorhabditis elegans*, SKN‐1 (Nrf2 homolog) has been implicated in the mechanism of metformin (Cabreiro et al., 2013; Onken & Driscoll, 2010; Wu et al., 2016); however, whether metformin can regulate the antioxidant response associated with human cellular aging and whether Nrf2 is involved in this process remain unanswered questions.

Mitochondria and the endoplasmic reticulum (ER) are two major organelles that contribute to ROS production in eukaryotic cells (Orrenius, Gogvadze, & Zhivotovsky, 2007). Although studies have reported that metformin regulates redox metabolism in mitochondria (Novelle, Ali, Dieguez, Bernier, & de Cabo, 2016), little is known about the effects of metformin on the redox signaling associated with the ER. In mammalian cells, there are three peroxidases located in the ER, namely glutathione peroxidase 7 (GPx7) (Wang, Zhang, Niu, Sitia, & Wang, 2014), glutathione peroxidase 8 (GPx8) (Ramming, Hansen, Nagata, Ellgaard, & Appenzeller‐Herzog, 2014), and peroxiredoxin 4 (Prx4) (Zito et al., 2010). Of these peroxidases, GPx7 plays a particularly crucial role as *GPX7*‐deficient cells display increased ROS levels and an accumulation of misfolded proteins (Peng et al., 2012; Wei et al., 2012); furthermore, *GPX7*‐knockout mice exhibit increased systemic oxidative stress, increased carcinogenesis, and shortened lifespan (Wei et al., 2012).

Here, we report that chronic low‐dose metformin treatment increases the lifespan of human diploid fibroblasts (HDFs) and human mesenchymal stem cells (HMSCs) through Nrf2‐mediated transcriptional upregulation of ER‐localized GPx7. Additionally, the geroprotection of the metformin‐Nrf2‐GPx7 pathway in aging is conserved in worms, suggesting that this beneficial pathway might be a general participant in pro‐longevity signaling.

## 2 RESULTS

### 2.1 Chronic low‐dose metformin treatment delays senescence in HDFs

**Figure 1:**
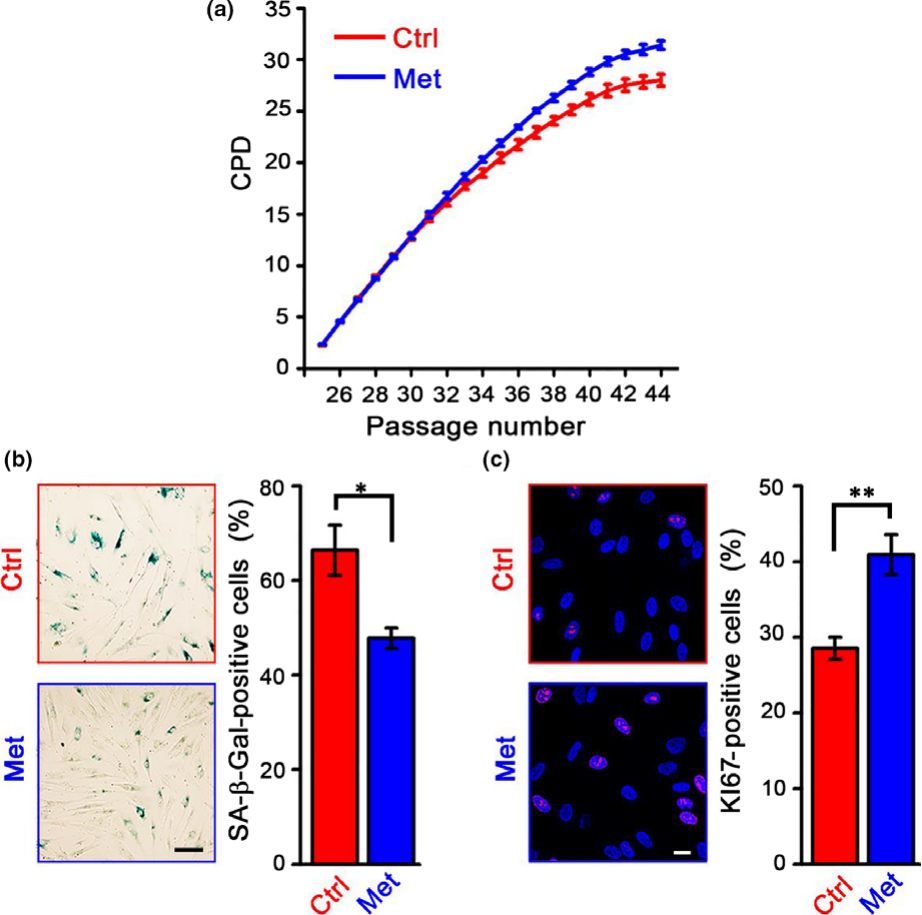
Metformin delays replicative senescence of human diploid fibroblasts (HDFs). (a) Cumulative population doubling (CPD) analysis of HDFs proliferation in the absence (Ctrl) or presence of 100 μm metformin (Met). (b) *Left*: senescence‐associated β‐galactosidase (SA‐β‐Gal) staining of HDFs at late passages (LP, P40–P45). Scale bar = 100 μm. *Right*: statistical analysis of the percentages of SA‐β‐Gal‐positive cells. (c) *Left*: KI67 expression in HDFs at LP. Scale bar = 20 μm. *Right*: statistical analysis of the percentages of KI67‐positive cells. Data were represented as mean ± *SEM* from three biological replicates, *n* > 200 cells per condition. *p <* *.05, **p <* *.01, via two‐tailed Student's *t* test

To determine the optimal concentration of metformin treatment for HDFs, we first assessed metformin cytotoxicity at various concentrations and found that up to 10 mm metformin did not compromise the cell apoptosis of HDFs within 24 hr (Fig. S1A). Next, to evaluate the long‐term effects of metformin treatment on HDFs, we cultured HDFs with metformin at dosages frequently used in cellular assays (1 and 10 mm) (Martin‐Castillo, Vazquez‐Martin, Oliveras‐Ferraros, & Menendez, 2010). Human diploid fibroblasts growth was impaired when the cells were cultured in medium containing 10 mm metformin (from passage 24 [P24] to P35) (Fig. S1B), suggesting that high‐dose metformin treatment might compromise the activity of HDFs. We did not observe significant changes when HDFs were treated with 1 mm metformin (from P24 to P44, Fig. S1B). We next investigated whether a lower dose of metformin treatment would hinder replicative senescence in HDFs. We observed that 100 μm metformin effectively stimulated HDF proliferation (Figure 1a), which was characterized by the reduced percentage of senescence‐associated β‐galactosidase (SA‐β‐Gal)‐positive cells; 100 μm metformin also increased the frequency of proliferation‐related KI67‐positive cells (Figure 1b,c). These results demonstrate that low‐dose metformin treatment exerets a geroprotective effect on HDFs.

### 2.2 GPx7 is a metformin target and regulates HDF senescence

**Figure 2:**
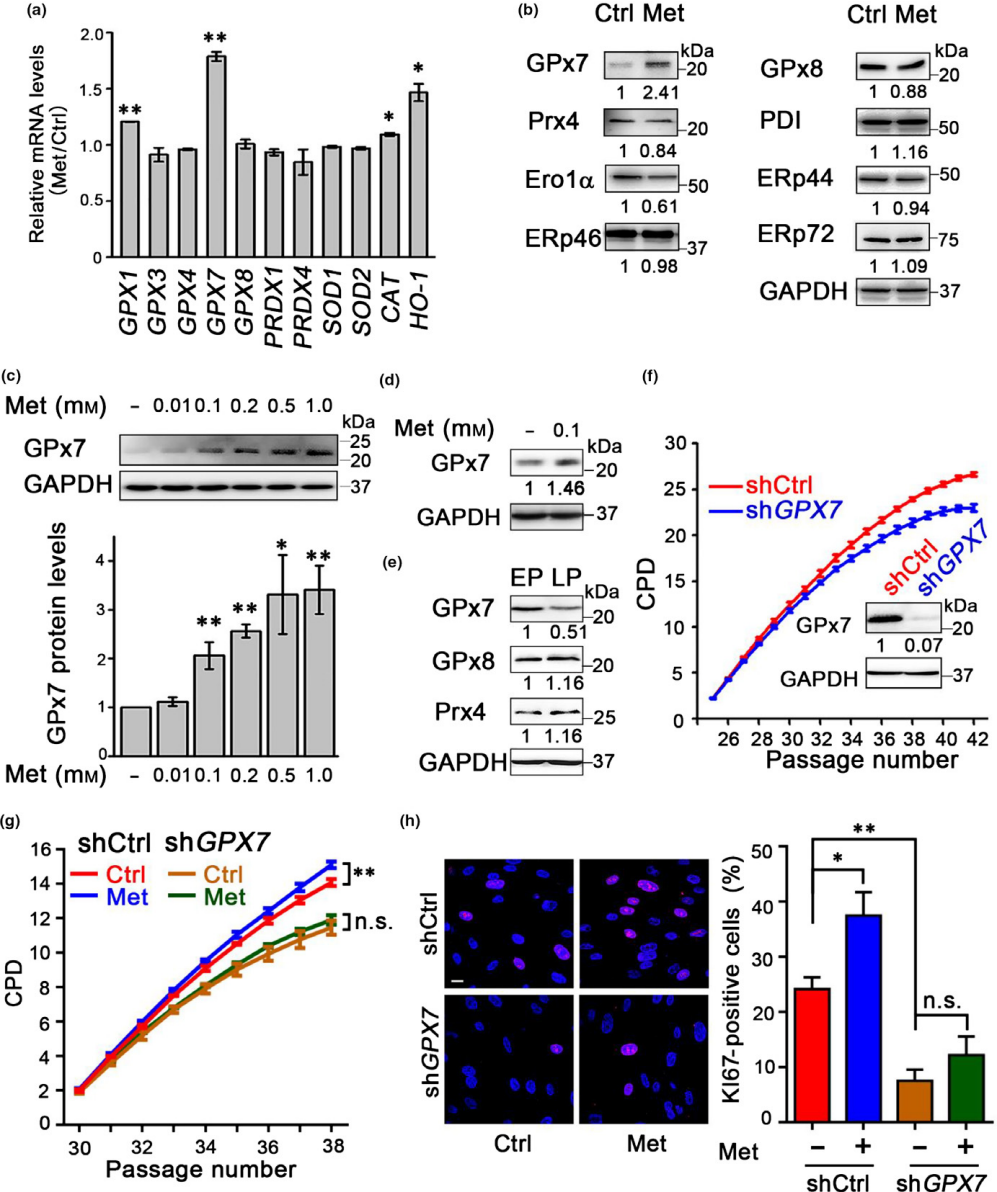
Glutathione peroxidase 7 (GPx7) is a key regulator in the senescence of human diploid fibroblasts (HDFs). (a) Relative mRNA level of redox‐related genes in HDFs (P30) induced by 200 μm metformin (Met) for 6 hr analyzed by RT–qPCR, normalized to control cells. Data were represented as mean ± *SEM* from three technical replicates, *p <* *.05, **p <* *.01, via two‐tailed Student's *t* test. Results are representative of three independent experiments. (b) Protein expression of ER‐localized oxidoreductases in HDFs (P30) treated without or with 200 μm metformin for 24 hr. (c) *Upper *:GPx7 in HDFs (P30) treated with indicated concentrations of metformin for 12 hr. *Lower*: statistical analysis of the expression of GPx7. Data were represented as mean ± *SEM* from four biological replicates. *p <* *.05, **p <* *.01, via two‐tailed Student's *t* test. (d) GPx7 in HDFs (P36) treated without or with 100 μm metformin since P25. (e) Expression of ER‐localized peroxidases in HDFs at both early passages (EP) and LP. (f) Cumulative population doubling (CPD) analysis of HDFs proliferation transduced with lentiviral shRNA control (shCtrl) and shRNA targeting *GPX7* (sh*GPX7*). *Inset*: GPx7 expression in HDFs transduced with lentivirus. (g) CPD analysis of shCtrl or sh*GPX7* HDFs in the absence (Ctrl) or presence of 100 μm metformin. Data were represented as mean ± *SEM* from three biological replicates, **p <* *.01, n.s., not significant, via two‐way ANOVA, Tukey's multiple comparisons test. (h) *Left*: KI67 expression in HDFs from (g) at P37. Scale bar = 20 μm. *Right*: statistical analysis of the percentages of KI67‐positive cells. Data were represented as mean ± *SEM* from three biological replicates, *n* > 200 cells per condition. *p <* *.05, **p <* *.01, n.s., not significant, via two‐way ANOVA, Tukey's multiple comparisons test. ER, endoplasmic reticulum

To investigate the mechanisms of the geroprotective effect of metformin on HDFs, we first investigated the most common signal pathway mediated by AMPK (Zhou et al., 2001). However, AMPK activation was not observed in HDFs treated with 100 μm metformin (Fig. S2). Because metformin has also been reported to be associated with cellular oxidative stress (Algire et al., 2012; Pernicova & Korbonits, 2014), we then examined the transcript levels of 11 antioxidant genes associated with oxidative stress (Li et al., 2015; Rhee, Woo, Kil & Bae, 2012; Turpaev, 2002) after 6 hr of metformin treatment. Of these genes, *GPX7* and *HO‐1 (HMOX1)* were the most strongly upregulated (Figure 2a), suggesting the potential involvement of these two genes in regulating aging and/or homeostasis in HDFs. As cytosolic heme oxygenase 1, encoded by the *HO‐1* gene, has been extensively investigated in regulating ROS metabolism (Gozzelino, Jeney, & Soares, 2010), we thus focused on GPx7, a less characterized ER‐localized peroxidase. Immunoblotting analysis identified that of the eight major oxidoreductases involved in redox regulation in the ER, GPx7 was the only one upregulated by metformin (Figure 2b). The positive effect of metformin on stimulating GPx7 expression was dose‐dependent and was confirmed in two independent HDF lines (Figure 2c and Fig. S3A–C). Moreover, GPx7 expression levels in HDFs were indeed increased throughout the passaging by metformin treatment (Figure 2d). Furthermore, we observed that GPx7 protein levels in HDFs decreased with serial passaging, whereas no significant changes in the two other ER‐localized peroxidases GPx8 and Prx4 were observed (Figure 2e). To determine whether GPx7 plays a role in regulating ER homeostasis and cellular aging, we knocked down *GPX7* in HDFs using lentiviral shRNA vectors (Figure 2f). Depleting *GPX7* induced features typical of cellular senescence (Figure 2f), including increased SA‐β‐Gal activity (Fig. S4A) and a decreased number of KI67‐positive cells (Fig. S4B). Importantly, depleting *GPX7* diminished the geroprotective effect of metformin (Figure 2g,h), underlying the genetic relationship between GPx7 and metformin. However, enforced expression of GPx7 is insufficient for extending proliferation in wild‐type cells (Fig. S4C), demonstrating a necessary but not sufficient role of GPx7 in geroprotection. Taken together, these results support the hypothesis that GPx7 is a target of metformin and that downregulating GPx7 during HDFs aging may contribute to cell growth arrest.

### 2.3 Metformin upregulates GPx7 in an Nrf2‐dependent manner

**Figure 3:**
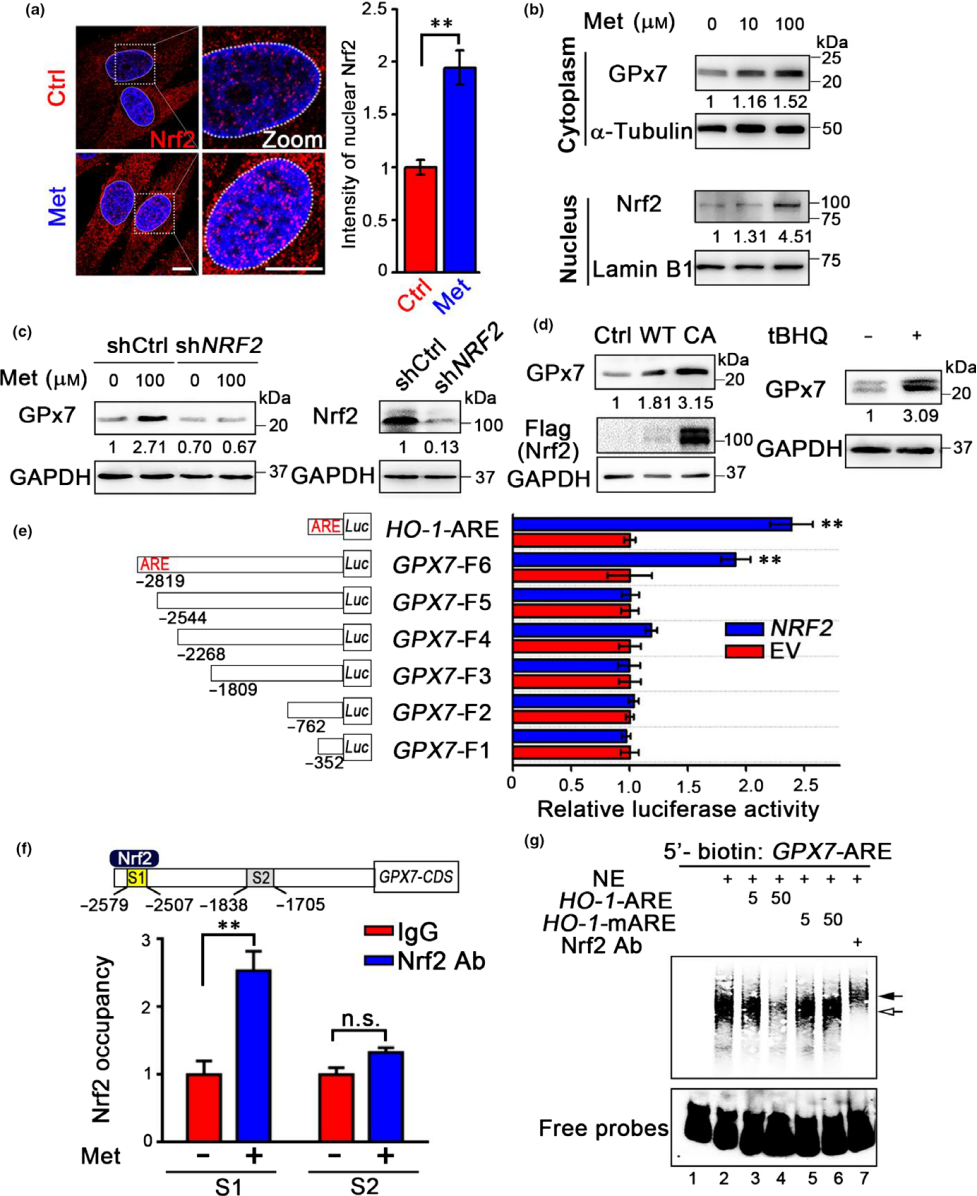
Metformin upregulates glutathione peroxidase 7 (GPx7) through activation of nuclear factor erythroid 2‐related factor 2 (Nrf2). (a) *Left*: immunofluorescence analysis of the translocation of Nrf2 to nucleus in human diploid fibroblasts (HDFs) treated with or without 100 μm metformin (Met) for 4 hr. Scale bar = 10 μm. The nuclei were outlined in dotted white circles. *Right*: statistical analysis of the fluorescence intensity of Nrf2 localized in the nucleus. Data were represented as mean ± *SEM*,* n* = 7 nuclei per condition. **p <* *.01, via two‐tailed Student's *t* test. Results are representative of two independent experiments. (b) Cytosolic GPx7 and nuclear Nrf2 in HDFs treated with indicated concentration of metformin for 6 hr. α‐Tubulin and Lamin B1 were used as cytoplasmic and nuclear loading controls, respectively. (c) *Left *:GPx7 expression in HDFs transduced with shCtrl or sh*NRF2* lentivirus, treated with or without 100 μm metformin for 12 hr; *right*: the efficiency of *NRF2* depletion. (d) *Left *:GPx7 protein levels in HDFs transduced with *GFP* (Ctrl), wild‐type *NRF2* (WT), or constitutively activated *NRF2* (CA) lentivirus; *Right *:GPx7 protein levels in HDFs stimulated without or with 200 μm tBHQ for 12 hr. (e) Luciferase reporter assay measuring *GPX7* promoter activity. HEK 293T cells were transfected with luciferase reporter construct (pGL3‐Basic) containing antioxidant response elements (ARE) of *HO‐1* or a series of 5′ fragments (F1–F6) of *GPX7* promoter as indicated, in combination with either pcDNA3.1‐*NRF2* or empty vector (EV). The relative luciferase activities were normalized relative to EV transfection. Data were represented as mean ± *SEM* from three technical replicates, **p <* *.01 via two‐tailed Student's *t* test. Results are representative of three independent experiments. (f) ChIP‐qPCR analysis of the Nrf2 occupancy on *GPX7* promoter. *Upper*: localization of the ARE‐containing sites (S1) and one nonspecific site (S2) at the *GPX7* promoter. *Lower*: ChIP‐qPCR analysis was performed with IgG or anti‐Nrf2 antibody in HDFs with or without 100 μm metformin treatment for 36 hr. Enrichment values were normalized to input and shown as the fold changes relative to IgG group. Data were represented as mean ± *SEM* from six technical replicates, **p <* *.01, n.s., not significant, via two‐way ANOVA, Tukey's multiple comparisons test. (g) Electrophoretic mobility shift assays (EMSA) analysis of the binding between Nrf2 and *GPX7*‐ARE. 5′‐biotin‐labeled *GPX7*‐ARE from S1 was incubated with HDFs nuclear extracts (NE) in the absence or presence of fivefold or 50‐fold excess of unlabeled *HO‐1*‐ARE or mutated *HO‐1*‐ARE (*HO‐1*‐mARE). The open arrow indicates the complex of endogenous Nrf2 with *GPX7*‐ARE, and the filled arrow indicates the ternary complex of Nrf2, *GPX7*‐ARE, and anti‐Nrf2 antibody.

We next examined the molecular mechanism by which metformin upregulates GPx7. As Nrf2 is a master transcription factor responsible for activating a variety of antioxidant genes, we hypothesized that metformin upregulates GPx7 through activating Nrf2. We noted that 100 μm metformin increased the nuclear accumulation of Nrf2 as well as the expression of GPx7 (Figure 3a,b). Metformin‐induced GPx7 expression was blocked after *NRF2* knockdown (Figure 3c). In addition, GPx7 was upregulated in HDFs overexpressing constitutively activated Nrf2 or treated with the Nrf2 activator tertiary butylhydroquinone (tBHQ) (Figure 3d). To identify whether any putative Nrf2‐binding AREs exist in the *GPX7* promoter, a series of luciferase reporter plasmids containing various truncated forms of the *GPX7* promoter were cotransfected with *NRF2* or an empty vector (Figure 3e). The Nrf2‐mediated activity was completely abolished when the region between −2819 and −2544 was absent (Figure 3e). This region contains the sequence TGACTTGGC, coinciding with the consensus ARE motif (TGACNNNGC). Chromatin immunoprecipitation–quantitative PCR (ChIP‐qPCR) of HDFs showed that this putative ARE associates with endogenous Nrf2 and this association was strengthened when the cells were treated with metformin (Figure 3f) and tBHQ (Fig. S5). Electrophoretic mobility shift assays (EMSA) indicated that *GPX7*‐ARE and endogenous Nrf2 in HDFs formed a slower migrating band, which was abrogated by excess unlabeled *HO‐1*‐ARE probes but not by mutated *HO‐1*‐ARE (Figure 3g). The binding specificity between *GPX7*‐ARE and Nrf2 was further confirmed by a supershift assay with an anti‐Nrf2 antibody (Figure 3g). The above results indicate that metformin induces *GPX7* expression in an Nrf2‐dependent manner, which may constitute a protective mechanism against HDF aging.

### 2.4 GPx7 plays an important role in defense against oxidative stress

**Figure 4:**
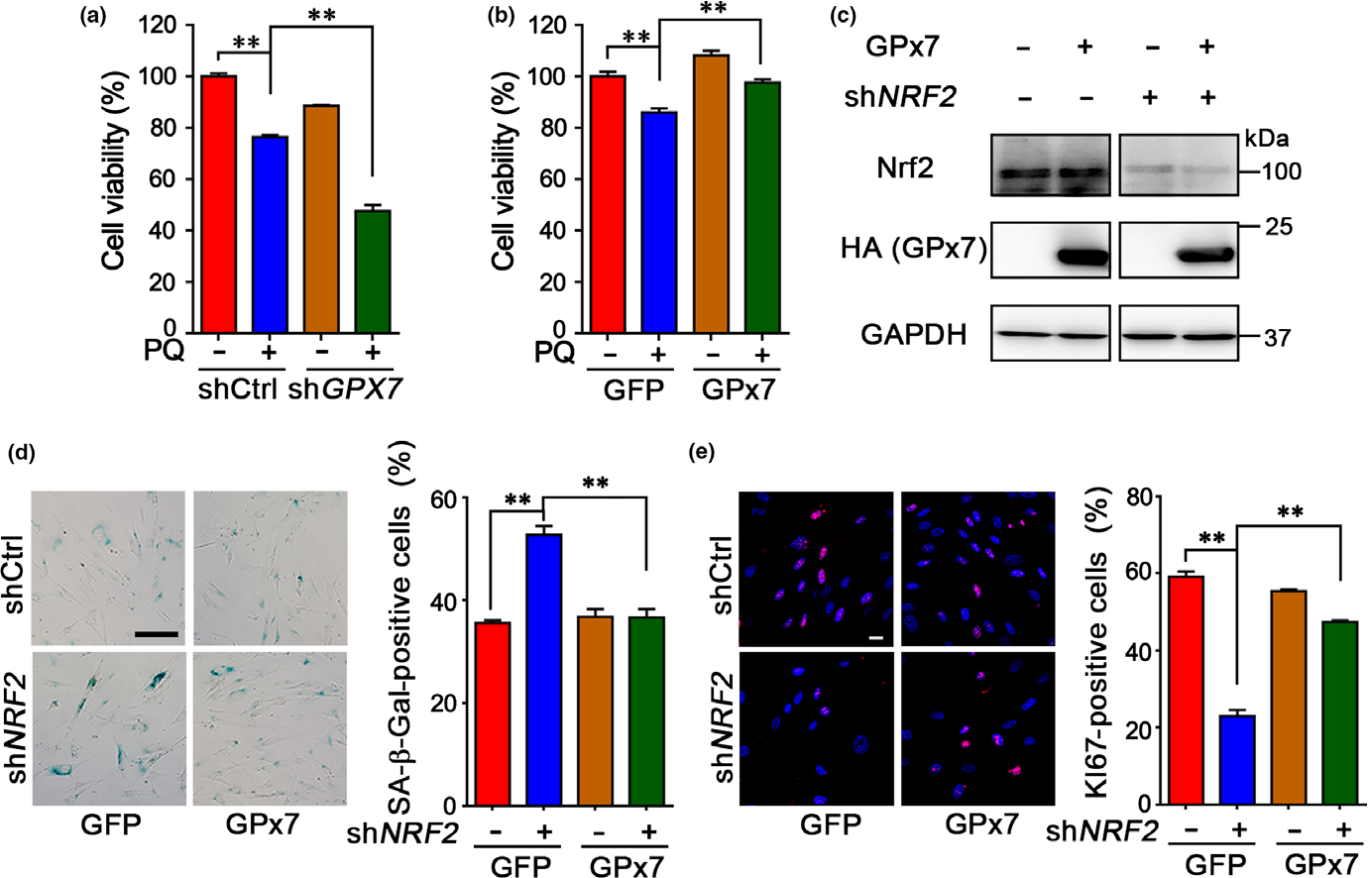
Glutathione peroxidase 7 (GPx7) plays a key role in defencing the oxidative stress in human diploid fibroblasts (HDFs). (a, b) Cell viability analysis of the proliferation of HDFs at P35 (a) or at P36 (b) in the absence or presence of 1 mm paraquat (PQ) for 24 hr. The HDFs were stably transfected with shCtrl*,* sh*GPX7*, GFP, GPX7 lentivirus as indicated. Data were represented as mean ± *SEM* from three technical replicates, **p <* *.01 via two‐way ANOVA, Tukey's multiple comparisons test. Results are representative of two independent experiments. (c) Western blotting to detect the efficiency of knockdown of nuclear factor erythroid 2‐related factor 2 (Nrf2) and overexpression of GPx7 by lentivirus as indicated in HDFs.(d, e) *Left*: SA‐β‐Gal staining (d) and KI67 expression (e) of HDFs at P28. Scale bar = 50 and 20 μm in (d) and (e), respectively. *Right*: Statistical analysis of the percentages of SA‐β‐Gal‐positive cells and KI67‐positive cells is illustrated. Data were represented as mean ± *SEM* from three biological replicates, *n* > 200 cells per condition,**p <* *.01 via two‐way ANOVA, Tukey's multiple comparisons test

To determine whether GPx7 regulates HDFs aging through its antioxidant activity, we first utilized paraquat as an oxidative stress inducer and observed that *GPX7* deficiency exacerbated paraquat‐induced oxidative stress damages in HDFs (Figure 4a); however, GPx7 overexpression provided HDFs with resistance to paraquat toxicity (Figure 4b). As Nrf2 impairment causes oxidative stress and recapitulates the progeroid phenotype (Kubben et al., 2016), we also examined the geroprotective effects of GPx7 overexpression on *NRF2*‐deficient HDFs. GPx7 overexpression alleviated the senescent phenotypes induced by *NRF2* depletion in two independent HDF lines (Figure 4c–e and Fig. S3D–F). The above results indicate that GPx7, as a downstream factor of Nrf2, plays an important role in defense against oxidative stress and aging.

### 2.5 The metformin‐Nrf2‐GPx7 pathway functions in HMSC aging

**Figure 5:**
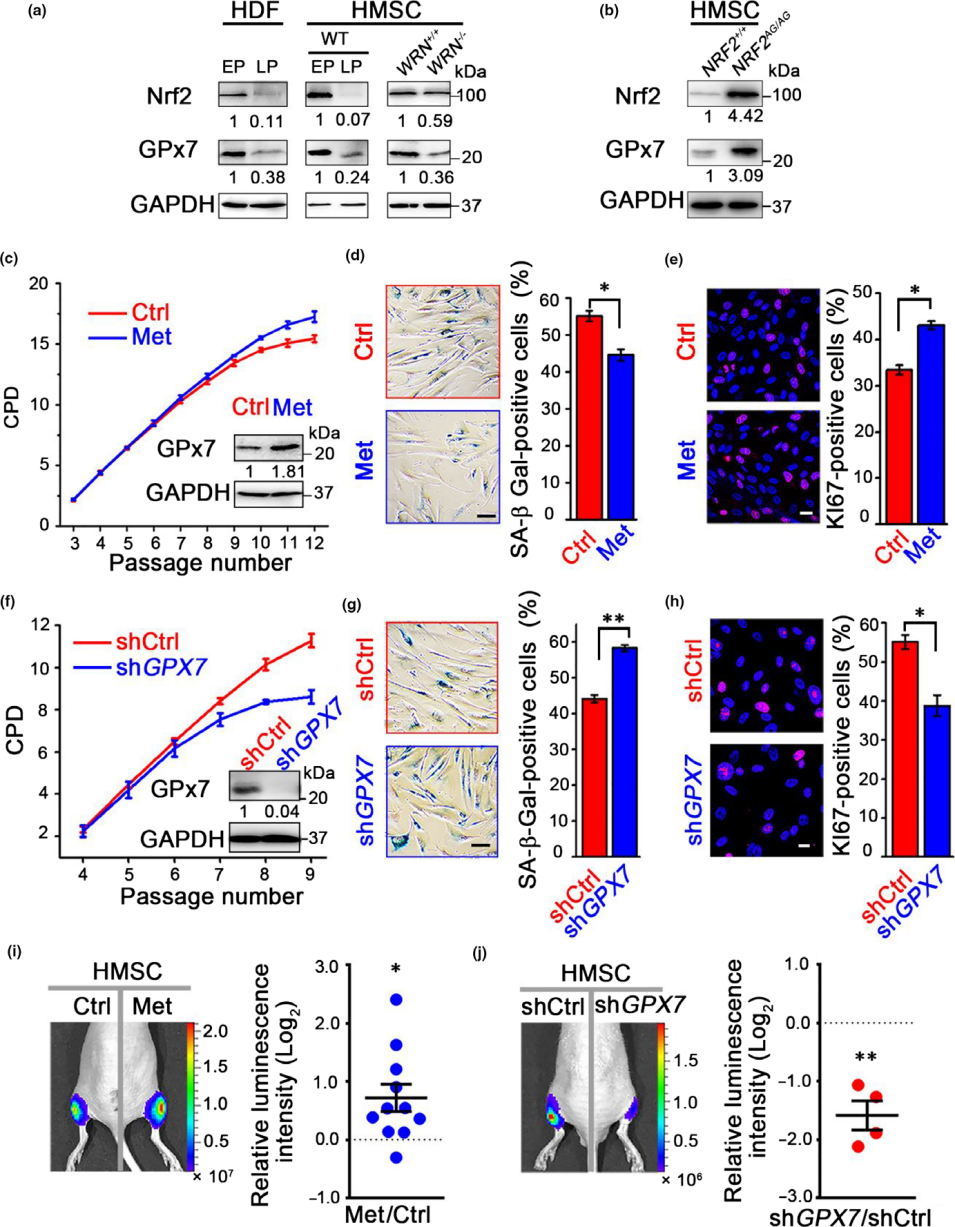
Metformin‐Nrf2‐GPx7 pathway functions in human mesenchymal stem cells (HMSCs). (a) Protein expression of nuclear factor erythroid 2‐related factor 2 (Nrf2) and glutathione peroxidase 7 (GP×7) in human diploid fibroblasts (HDFs) and HMSCs at EP and LP, and in *WRN* ^+/+^ and *WRN* ^−/−^ HMSCs at P5. For HMSCs, P3–P5 are taken as EP and P9–P12 as LP. (b) Nrf2 and GPx7 in wild‐type HMSCs (*NRF2* ^*+/+*^) and HMSCs genetically bearing an endogenous *NRF2* (A254G) variation (*NRF2* ^*AG*^ ^*/*^ ^*AG*^). (c, f) Cumulative population doubling (CPD) analysis of HMSCs proliferation. Wild‐type HMSCs from P3 to P12 were continuously cultured in the absence or presence of 100 μm metformin (Met) (c). *Inset*: the expression of GPx7 at P7. HMSCs transduced with shCtrl and sh*GPX7* lentivirus were continuously cultured from P4 to P9 (f). *Inset *:GPx7 expression in HMSCs transduced with lentivirus. (d, g) *Left *:SA‐β‐Gal staining of wild‐type HMSCs at LP (d) and lentivirus‐transduced HSMCs at P7 (g), respectively. Scale bar = 100 μm. *Right*: statistical analysis of the percentages of SA‐β‐Gal‐positive cells. (e, h) *Left *:KI67 expression in wild‐type HMSCs at LP (e) and lentivirus‐transduced HMSCs at P7 (h), respectively. Scale bar = 20 μm. *Right*: statistical analysis of the percentages of KI67‐positive cells. Data were represented as mean ± *SEM* from three biological replicates, *n* > 200 cells per condition. *p <* *.05, **p <* *.01, via two‐tailed Student's *t* test. (i, j) Measurement of the in vivo retention of transplanted HMSCs by in vivo imaging system. *Left*: Conditioned HMSCs with serial metformin administration (i) or transduced with shRNA lentivirus (j) overexpressing luciferase were implanted into the left and right tibialis anterior muscles of immune‐deficient mice, respectively. Photon flux was captured on the fifth or sixth day after implantation. *Right*: Statistical analysis of each mouse implanted with HMSCs with the relative luminescence intensities normalized to log_2_ fold. Data were presented as scatter dot plots displaying the mean ± *SEM*, from eleven (i) or four (j) biological replicates. *p <* *.05, **p <* *.01, via two‐tailed Student's *t* test.

Stem cell exhaustion causes organismal aging and contributes to various aging‐associated disorders (Oh, Lee, & Wagers, 2014). We next investigated whether the metformin‐Nrf2‐GPx7 pathway also functions in HMSCs. We observed that the expression levels of Nrf2 and GPx7 decreased in both replicative senescent HMSCs and premature senescent HMSCs (Werner syndrome‐specific: *WRN*‐deficient) (Li et al., 2016; Zhang et al., 2015) (Figure 5a). High levels of GPx7 were also observed in HMSCs genetically bearing an endogenous *NRF2* nucleotide variation (A254G, referred to as HMSC‐*NRF2* ^*AG/AG*^) (Yang et al., 2017) (Figure 5b), which encodes a constitutively activated Nrf2 protein (Fig. S6) and confers an extended lifespan. As in HDFs, chronic low‐dose metformin treatment alleviated the senescence features of HMSCs (Figure 5c–e). Knocking down *GPX7* in wild‐type HMSCs resulted in accelerated cellular aging (Figure 5f–h). To investigate whether the metformin‐Nrf2‐GPx7 pathway could protect HMSCs in an in vivo context, HMSCs were implanted into the tibialis anterior muscle of immune‐deficient mice, and their in vivo retention was measured by an in vivo imaging system (IVIS) (Kubben et al., 2016; Pan et al., 2016; Wang et al., 2018; Yang et al., 2017). Metformin‐treated HMSCs displayed delayed cellular attrition compared to vehicle‐treated cells (Figure 5i), whereas *GPX7*‐deficient HMSCs exhibited accelerated cell exhaustion in the in vivo microenvironment (Figure 5j). Based on these findings, we conclude that the metformin‐Nrf2‐GPx7 pathway safeguards HMSCs from premature aging.

### 2.6 Metformin delays *Caenorhabditis elegans* aging through the conserved SKN‐1‐GPX‐6 pathway

**Figure 6:**
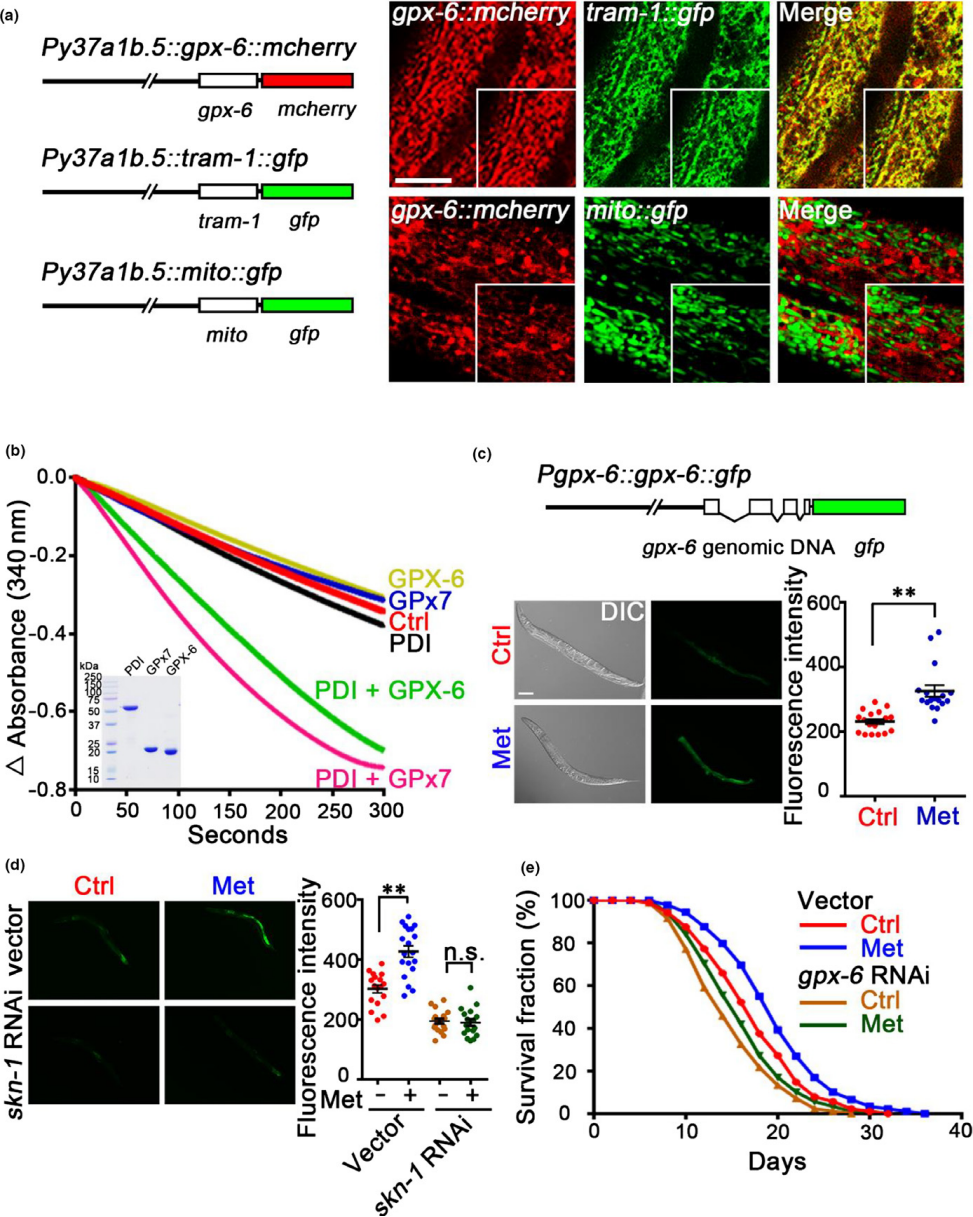
The Metformin‐SKN‐1‐GPX‐6 pathway functions in *Caenorhabditis elegans*. (a) *Left: mcherry* was translationally fused to the CDS encoding *gpx‐6* and *gfp* was translationally fused to the genomic DNA encoding *tram‐1* or mitochondrial signal peptide (mito), respectively. The expression was driven by the *y37a1b.5* promoter. *Right*: Transgenic worms simultaneously expressed GPX‐6 [*Py37a1b.5::gpx‐6::mcherry*] (red channel) and TRAM‐1 (upper) [*Py37a1b.5::tram‐1::gfp*] (green channel) or Mito‐GFP (lower) [*Py37a1b.5::mito::gfp*]. Scale bar = 5 μm. (b) Human glutathione peroxidase 7 (GPx7) and *C. elegans* GPX‐6 peroxidase activities were measured by the decrease in absorbance at 340 nm due to NADPH consumption. *Inset*: purification of the recombinant human PDI, human GPx7, and *C. elegans* GPX‐6. (c) *Upper: gfp* was translationally fused to the full‐length genomic DNA of *gpx‐6* including the 2,876‐bp promoter regions. *Lower*: Images and quantification of the worms expressing GPX‐6::GFP subjected to vehicle (Ctrl) or 50 mm metformin (Met) for 24 hr post‐L4 larval stage. Scale bars = 100 μm. Data were represented as mean ± *SEM*,* n* ≥ 14 worms per condition. **p <* *.01, via one‐way ANOVA, Tukey's multiple comparisons test. (d) Worms expressing GPX‐6::GFP without or with *skn‐1* RNAi were subjected to 50 mm metformin for 48 hr post‐L4 larval stage, imaged, and quantified. Scale bars = 100 μm. Data were represented as mean ± *SEM*,* n* > 15 worms per condition. **p <* *.01 via two‐way ANOVA, Tukey's multiple comparisons test. Results are representative of two independent experiments. (e) Survival curves of WT or *gpx‐6* RNAi worms treated without or with 50 mm metformin. Results are representative of four independent experiments. The mean lifespan is 16.1 and 18.7 days for vector fed worms raised on 0 and 50 mm metformin, respectively, and the survival curves of the two groups are significantly different (*p *=* *.0012 via the log‐rank test). The mean lifespan is 13.8 and 14.6 days for *gpx‐6* RNAi fed worms raised on 0 and 50 mm metformin, respectively, and the survival curves of the two groups are not significant (*p *=* *.2455 via the log‐rank test) (see also Table S1).

Finally, we investigated whether the metformin‐Nrf2‐GPx7 axis also functions in organism aging using *C. elegans* as a model. There are eight glutathione peroxidases (GPX‐1–GPX‐8) in *C. elegans*; GPX‐6 and GPX‐7 possess a predicted ER signal peptide and a potential ER retention motif, respectively (Fig. S7A,B). To confirm whether these two GPX proteins were located in the ER like human GPx7, we generated *Py37a1b.5::gpx‐6::mcherry* and *Py37a1b.5::gpx‐7::mcherry* and examined their localization in the hypodermal cells. As shown in Figure 6a, GPX‐6::mCherry demonstrated a marked colocalization with the ER‐marker TRAM1::GFP but did not match the outlines of mitochondria. By contrast, GPX‐7::mCherry did not demonstrate a marked ER localization pattern (Fig. S7C). To further confirm that GPX‐6 in *C. elegans* and GPx7 in human share functional similarities, we purified both proteins and measured their peroxidase activities by in vitro NADPH consumption assays. A unique feature of human GPx7 is that it uses H_2_O_2_ to oxidize the ER‐localized protein disulfide isomerase (PDI) rather than glutathione itself (Nguyen et al., 2011; Wang et al., 2014). Like human GPx7, *C. elegans* GPX‐6 did not display any glutathione peroxidase activity but had greatly increased peroxidase activity when PDI was used as a substrate (Figure 6b). Thus, we determine *C. elegans* GPX‐6 to be the ortholog of human GPx7.

To determine whether GPX‐6 is regulated by metformin, we generated the reporter *Pgpx‐6::gpx‐6::gfp* with full‐length genomic DNA of *gpx‐6*, including its own 2,876‐bp promoter regions; we observed that GPX‐6 was upregulated by metformin in *C. elegans* (Figure 6c) and was also induced by tBHQ (Fig. S8A). Moreover, *skn‐1* RNAi in *C. elegans* completely abrogated the induction of GPX‐6 by metformin (Figure 6d), suggesting that metformin upregulates GPX‐6 expression mainly through SKN‐1 in worms. Similar to the results obtained in human cells, GPX‐6 expression in worms was decreased during aging (Fig. S8B), and *gpx‐6* RNAi (Fig. S8C) increased the sensitivity of the worms to oxidative stress (Fig. S8D) and shortened the mean lifespan of the worms (Figure 6e). Interestingly, although metformin significantly extended the mean lifespan of the worms, GPX‐6 was required for these metformin‐mediated effects (Figure 6e and Table S1), suggesting that GPX‐6 functions in the positive effects of metformin on lifespan extension.

## 3 DISCUSSION

Over the past decades, scientific studies on aging have demonstrated that genetic modulation can extend lifespan in diverse model organisms and have established that aging can be targeted by dietary and pharmacologic interventions (Longo et al., 2015). Metformin, the most widely used antidiabetic drug in the world, has been reported to favorably influence metabolic and cellular processes closely associated with the development of aging and even to delay aging in animal models (Novelle et al., 2016). However, there is still insufficient evidence to prove the geroprotective effects of metformin in normal human diploid cells. In this work, by monitoring the replicative senescence of both HDFs and HMSCs, we provide strong evidence that chronic low‐dose metformin treatment can delay human cellular aging. Our findings on the geroprotective effects of low‐dose metformin treatment on human diploid cells may contribute to the TAME project and its related clinical application.

Notably, the major aging‐suppressing effects of metformin in animal models have been achieved at doses higher than the therapeutic doses given to diabetic patients, whose plasma concentrations of metformin are usually <50 μm (Martin‐Castillo et al., 2010). We found that a high dose of metformin (10 mm) results in reduced HDFs proliferation, which has also been reported elsewhere (Menendez et al., 2011). Interestingly, chronic treatment with 100 μm metformin, comparable to the plasma concentrations in metformin‐treated diabetic patients, suppressed HDF and HMSC senescence. Very recently, it was reported that 500 μm metformin upregulates DICER1 expression to decrease cellular senescence in stress‐induced senescence models (Noren Hooten et al., 2016). It is likely that metformin at different concentrations impacts different signaling pathways in human cells. The observation that metformin activates the Nrf2 pathway at a low concentration but stimulates AMPK phosphorylation at only millimolar levels (Fig. S2) suggests that the geroprotective effects of a low dose of metformin on human mesodermal cells might be independent of the AMPK pathway. Many studies of the AMPK/mTOR‐dependent anticancer effects of metformin were carried out using concentrations between 5 and 10 mm (Martin‐Castillo et al., 2010); such concentrations, however, result in diminished HDF activity. Coincidently, a similar detrimental phenotype has been observed in metformin‐treated mouse models; chronic administration of a low dose of metformin (0.1% w/w in the diet) improved the health and lifespan of mice, while a higher dose (1% w/w) was toxic (Martin‐Montalvo et al., 2013). Although we treated worms with 50 mm metformin, *C. elegan*s has a highly protective cuticle and intestine that generally limit drug uptake; polar drugs, such as metformin, can be applied at a concentration approximately 1,000‐fold higher than their predicted affinity for the target (Holden‐Dye, & Walker, 2007). Nevertheless, it should be considered that under these culture conditions, AMPK‐dependent activation of SKN‐1 could also contribute to metformin‐induced lifespan extension in *C. elegans* (Cabreiro et al., 2013; Onken & Driscoll, 2010).

**Figure 7:**
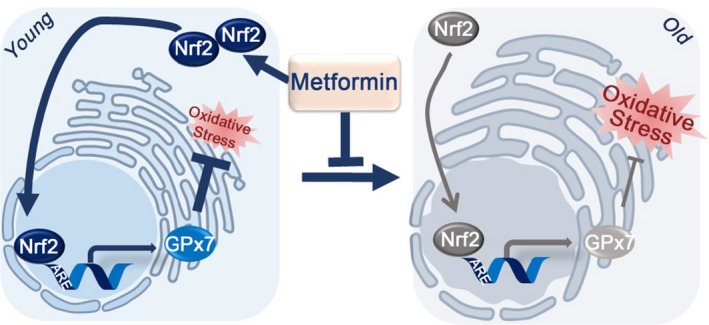
A model illustrating metformin delays aging through the Nrf2‐GPx7 pathway. Nuclear factor erythroid 2‐related factor 2 (Nrf2) is a master transcription factor for modulating cellular antioxidant responses. Through binding onto the antioxidant response elements (ARE), Nrf2 stimulates the expression of a wide arrays of antioxidant enzymes, among which glutathione peroxidase 7 (GPx7) is a unique ER‐localized peroxidase. In young cells, sufficient Nrf2 transcriptionally induces GPx7 expression to defend oxidative stress. In old cells, Nrf2 and GPx7 expression decreases and oxidative stress accumulates. Low‐dose metformin can promote the nuclear translocation of Nrf2 to upregulate the expression of GPx7 to alleviate cellular aging. ER, endoplasmic reticulum

As the beneficial effects of low‐dose metformin treatment on human mesodermal cells occur mainly through the Nrf2 pathway, we focused on the antioxidant role of metformin. Metformin has been reported to reduce oxidative stress and thus DNA damage (Algire et al., 2012). Our results show that chronic metformin treatment upregulates an ER‐localized peroxidase, GPx7, which safeguards worms and human cells from premature aging. We further reveal that the upregulation of GPx7 by metformin occurs through the Nrf2‐ARE axis, and we identify a functional ARE in the promoter of *GPX7* (Figure 7). Also, metformin activates the Nrf2‐dependent antioxidant response in worm (Onken & Driscoll, 2010) and mouse (Martin‐Montalvo et al., 2013) models. The unresolved question is how metformin activates Nrf2. One possibility is that metformin induces mitohormesis through perturbing the mitochondrion respiratory chain (De Haes et al., 2014; Foretz et al., 2010). However, very high concentrations of metformin (5 mm) were required to observe significant inhibition of mitochondrion respiratory chain complex 1 activity (He & Wondisford, 2015). Recent studies showed that Nrf2 can be activated by the ER unfolded protein response sensors IRE1α (Hourihan, Moronetti Mazzeo, Fernandez‐Cardenas, & Blackwell, 2016) and PERK (Cullinan et al., 2003). However, in our hands, PERK activation was not detected when HDFs were treated with 100 μm metformin (Fig. S9). At this stage, the mechanism by which metformin activates Nrf2 remains unknown. Emerging evidence shows that oxidative protein folding in the ER is one of the main sources of cellular ROS production (Delaunay‐Moisan, & Appenzeller‐Herzog, 2015; Konno et al., 2015), and that ER redox was perturbed throughout the lifespan of *C*. *elegans* (Kirstein et al., 2015). Thus, it will be interesting to determine whether Nrf2 can regulate *GPX7* to maintain ER homeostasis by sensing the redox signals initiating from the ER during aging.

## 4 EXPERIMENTAL PROCEDURES

### 4.1 Cell culture

HDFs (AG07095, from Coriell Cell Repository) were cultured in DMEM (Gibco, 11995‐065) supplemented with 10% fetal bovine serum (Gibco, 10099‐141), 0.1 mm nonessential amino acids (Gibco), 1% penicillin/streptomycin (Gibco). HMSCs were differentiated from H9 human embryonic stem cells (WiCell Research) based on a published protocol (Liu et al., 2014) and were cultured in MEMα (Gibco, 41096‐036) supplemented with 10% FBS, 0.1 mm nonessential amino acids, 1% penicillin/streptomycin, and 1 ng/ml basic fibroblast growth factor (Joint Protein Central).

### 4.2 Lentivirus preparation

The cDNA of Flag*‐NRF2,* Flag*‐NRF2* CA (E82G) and GFP were cloned into pLE4 lentiviral vector (a gift from Dr Tomoaki Hishida). The shRNA sequences (sh*GPX7*: GCAGGACTTCTACGACTTCAA, sh*NRF2*: GTAAGAAGCCAGATGTTAA) targeting *GPX7* and *NRF2* were cloned into pLVTHM/GFP (Addgene, 12247). For lentivirus packaging, HEK 293T cells were cotransfected with lentiviral vectors and packaging plasmids psPAX2 (Addgene, 12260) and pMD2G (Addgene, 12259) using Lipofectamine 2000 (Life Technologies). Lentivirus particles were collected on day 2, concentrated by ultracentrifugation at 19,400 *g* for 2.5 hr, and then used for transduction in the presence of 4 μg/ml polybrene.

### 4.3 Population doubling assay

HDFs were plated at 2 × 10^5^ per well in a six‐well plate, and HMSCs were plated at 1 × 10^5^ per well in a 12‐well plate. When cells reached 80%–95% confluent, serial passaging was performed and the number of cells was counted. Population doubling per passage was calculated as log_2_ (number of cells obtained/number of cells plated). When the number of the obtained cells is no more than the inoculated cells in 2 weeks, the cells were regarded as senescence. Cumulative population doublings of the cells were calculated and plotted to the passage numbers after lentivirus infection or PBS/metformin treatment.

### 4.4 SA‐β‐Gal staining assay

SA‐β‐Gal assays were carried out using Senescence β‐Galactosidase Staining Kit (Beyotime) as per the manufacturer's instructions. Briefly, cultured cells were washed in PBS and fixed at room temperature for 15 min in 4% formaldehyde and 0.2% glutaraldehyde. Fixed cells were stained with fresh staining solution for SA‐β‐Gal activity at 37°C for 14–18 hr. The percentage of cells positive for SA‐β‐Gal staining were quantified and statistically analyzed.

### 4.5 Western blotting

Cells were lysed using lysis buffer (Millipore) with Protease Inhibitor Cocktail (Roche). Protein quantification was performed using a BCA Kit (Beyotime). Protein lysate was subjected to SDS‐PAGE and subsequently electrotransferred onto a polyvinylidene fluoride membrane (Millipore). Blots were developed by indicated antibodies and enhanced chemiluminescence (ECL) (Millipore, WBKLS0500), followed by a ChemiScope Mini chemiluminescence imaging system (Clinx Science). The antibodies used are listed as follows: anti‐GPx7 (Abclone, A3902, 1:1,000), anti‐Nrf2 (Abcam, ab62352, 1:1,000), anti‐PDI (Abcam, ab2792, 1:2,000), anti‐GPx8 (GeneTex, GTX125992, 1:1,000), anti‐Prx4 (Animal Facility, Institute of Genetics and Developmental Biology, CAS, rabbit serum, 1:500), anti‐ERp44 (CST, 3798, 1:2,000), anti‐Ero1α (Millipore, MABT376, 1:1,000), anti‐ERp46 (Animal Facility, Institute of Genetics and Developmental Biology, CAS, rabbit serum, 1:500), anti‐ERp72 (Origene, TA503904, 1:2,000), anti‐GAPDH (Sigma, G9295, 1:50,000), anti‐Flag (Sigma, F1804, 1:4,000), anti‐α‐tubulin (Sigma, T6074, 1:10,000), goat anti‐rabbit IgG (Sigma, A0545, 1:10,000), goat anti‐mouse IgG (Sigma, A4416, 1:10,000).

### 4.6 Immunofluorescence

HDFs and HMSCs were fixed with formaldehyde (4% in PBS) for 15 min, permeabilized with Triton X‐100 (0.3% in PBS) for 15 min, incubated with BSA (3% in PBS) for 30 min, and stained with primary antibody for 1 hr at room temperature. The cells were then incubated with secondary antibodies for 1 hr at room temperature. Hoechst 33258 (Sigma) was used to stain nuclear DNA. The antibodies used in immunofluorescence assay are as follows: anti‐KI67 (Vector, VP‐RM04, 1:1,000), anti‐Nrf2 (Abcam, ab62352, 1:250), goat anti‐rabbit Alexa Fluor 488 (Life Technologies, A11034, 1:1,000), goat anti‐rabbit Alexa Fluor 568 (Life Technologies, A21069, 1:1,000).

### 4.7 RT–qPCR

Total cellular RNA was isolated using TRIzol reagent (Life Technologies). RNA samples (2 μg each) were then reverse‐transcribed into cDNA using GoScript Reverse Transcription System (Promega). Quantitative real‐time PCR was carried out using SYBR Select Master Mix (Applied Biosystems) and QuantStudio 7 Flex machine (Applied Biosystems), following the manufacturer's instructions. The primers used are listed in Appendix Table S2, and the relative levels of each gene expression were normalized to GAPDH and calculated as 2^−ΔΔCT^.

### 4.8 Dual‐luciferase reporter assay

The putative promoter regions of the human *GPX7* were isolated via PCR from genomic DNA of HDFs using TransStart FastPfu Fly DNA Polymerase (TransGen Biotech), and inserted into Kpn I/Xho I sites of the firefly luciferase reporter plasmid pGL3‐Basic (Promega). The primers used for the construction are listed in Appendix Table S3. All the constructs were confirmed by DNA sequencing; 1 × 10^5^ HEK 293T cells were seeded into 24‐well plates and transfected at 60% confluency using Viafect (Promega). Two hundred nanogram of each firefly luciferase reporter plasmid plus 40 ng of pRL‐TK (Promega) plasmid containing renilla reporter as the control were cotransfected with 500 ng pcDNA3.1‐*NRF2* or pcDNA3.1 empty plasmid. Luciferase activity was then measured 48 hr after transfection using the Dual‐Luciferase Reporter Assay System (Promega) with a GloMax Luminometer (Promega). Firefly luciferase activity was normalized to renilla luciferase activity for each transfected cell sample. The specific procedure was performed according to a published paper (Duan et al., 2015).

### 4.9 Subcellular fractionation

The cells were lysed with cytoplasmic lysis buffer (10 mm HEPES buffer, pH 7.9, containing 10 mm KCl, 1.5 mm MgCl_2_, 1 mm dithiothreitol [DTT], 0.4% [v/v] NP‐40 and Protease Inhibitor Cocktail), and the supernatant was collected by centrifugation at 10,000 *g* for 3 min. The intact nuclei were pelleted by centrifugation at 6,000 *g* for 3 min and washed twice with cytoplasmic lysis buffer without Protease Inhibitor Cocktail. Nuclei were then lysed with nuclear lysis buffer (20 mm HEPES buffer, pH 7.9, containing 0.2 mm EDTA, 0.1 mm EGTA, 420 mm NaCl, 25% [v/v] glycerol, 1 mm DTT, 0.1% [v/v] NP‐40 and Protease Inhibitor Cocktail). After vortex for 30 min on ice, the supernatant was collected by centrifugation at 12,000 *g* for 15 min as the nuclear fraction.

### 4.10 ChIP‐qPCR assay

ChIP‐qPCR was performed according to a previous protocol (Dahl & Collas, 2008; Yang et al., 2017) with slight modifications; 1 × 10^6^ HDFs with or without 100 μm metformin or 200 μm tBHQ treatment were cross‐linked in 1% v/v formaldehyde/PBS for 15 min at room temperature and then quenched by 125 mm Glycine. Samples were lysed on ice for 5 min. Subsequently, lysates were sonicated using a diagenode bioruptor with 8 × 30 s run plus 30 s pause. The collected supernatants were incubated overnight with Protein A Dynabeads (Life Technologies, 10001D) bound with 2.4 μg anti‐Nrf2 antibody (Abcam, ab62352) or rabbit IgG (Santa Cruz, SC‐2027). Next, the input sample and chromatin‐beads complexes were digested, eluted, and cross‐link‐reversed at 68°C for 2 hr on a thermomixer. DNA was finally purified by phenol–chloroform–isoamyl alcohol and chloroform–isoamyl alcohol extraction. The enriched DNA was further used for qPCR to detect the putative ARE of *GPX7*. The primers used for the enrichment are listed in Appendix Table S4.

### 4.11 Electrophoretic mobility shift assay

Synthesized forward and reverse strand oligonucleotides of the putative ARE in *GPX7* promoter region were hybridized to form double‐stranded DNA probes. The 5′ of forward strand was labeled by biotin. Wild‐type and mutant HO1‐ARE without biotin label were used as the competitor. Binding reactions were carried out using Chemiluminescent EMSA Kit (Beyotime) according to the manufacturer's instructions. For supershift analysis, extracts were pre‐incubated for 20 min on ice with anti‐Nrf2 antibody (Abcam, ab62352). DNA‐binding protein complexes were separated by nondenaturing 5% PAGE in 0.5× Tris‐Borate‐EDTA buffer and subsequently electrotransferred onto a positively charged nylon transfer membrane (GE Healthcare). Blots were developed by horseradish peroxidase‐labeled streptavidin and BeyoECL Star (Beyotime). All the sequences designed for EMSA are listed in Appendix Table S5.

### 4.12 Cell viability measurement

Cells were seeded into 96‐well plates at a density of 5, 000 cells per well, and paraquat treatment was initiated when the cells were about 90% confluent. After 24hr treatment, the viability measurement was carried out with the CellTiter 96 AQueous One Solution Cell Proliferation Assay kit (Promega) according to the manufacturer's instructions.

### 4.13 HMSCs transplantation assay

A total volume of 100 μl PBS of 1 × 10^6^ HMSCs labeled with luciferase were injected into the midportion of the tibialis anterior muscle of immune‐deficient BALB/c nude mice (Pan et al., 2016); 5 or 6 days after implantation, mice were anesthetized and treated with D‐luciferin. Then, photon emission was measured by the IVIS Lumina System (PerkinElmer). Bioluminescence images were acquired at auto‐set model. Photons were counted according to the digital false‐color photon emission image of the mouse, and the values were normalized by average cellular luciferase intensity before implantation. Animal experiments were conducted with the approval of the institutional committee of Institute of Biophysics, Chinese Academy of Science.

### 4.14 In vitro peroxidase activity assay

Mature human GPx7 and *C. elegans* GPX‐6 proteins including an N‐terminal 6× His tag were purified with a nickel‐chelating column (GE Healthcare), and peroxidase activity was conducted as previously described (Wang et al., 2014). In brief, the decrease in absorbance at 340 nm due to NADPH (150 μm; Roche) consumption by glutathione reductase (0.24 U; Sigma) was monitored, in the presence of 150 μm H_2_O_2_, 0.5 mm GSH (Sigma), and 10 μm human PDI, with or without 10 μm human GPx7 or *C. elegans* GPX‐6, respectively. All experiments were performed in 100 mm Tris‐HAc (pH 8.0) containing 50 mm NaCl and 1 mm EDTA at 25°C.

### 4.15 *Caenorhabditis elegans* strains

Strains were cultured at 20°C using standard methods. The following strains were used in this work: N2 Bristol (wild‐type), *bpEx272* [*Pgpx‐6::gpx‐6::gfp*], *bpEx273* [*Py37a1b.5::gpx‐6::mcherry; Py37a1b.5::tram‐1::gfp; rol‐6(su1006)*], *bpEx289* [*Py37a1b.5::gpx‐7::mcherry; Py37a1b.5::tram‐1::gfp; rol‐6(su1006)*], *bpEx334* [*Py37a1b.5::gpx‐6::mcherry; Py37a1b.5::mito::gfp; rol‐6(su1006)*].

### 4.16 RNAi in *Caenorhabditis elegans*

Animals were synchronized by a hypochlorite/sodium hydroxide egg preparation and placed on RNAi plates containing HT115(DE3) bacteria specific for *gpx‐6*,* skn‐1*, or the empty vector L4440 from the Ahringer library.

### 4.17 Lifespan assay for *Caenorhabditis elegans*

Lifespan analysis was conducted at 20°C according to a protocol modified from previous publication (Cabreiro et al., 2013; Wu et al., 2016). Briefly, synchronized L1 animals were seeded onto the standard nematode growth media (NGM) plates until L4 stages. On day 0, 20–40 L4 worms per plate (three to six plates, more than 100 worms in total per condition) were transferred onto RNAi plates with or without metformin cotreatment. All plates were supplemented with 15 μm 5‐fluorodeoxyuridine (FUdR) solution to suppress progeny production. The mean lifespan was calculated with online OASIS2 resources (Han et al., 2016).

### 4.18 Statistical analysis

Results were presented as mean ± *SEM*. Two‐tailed Student's *t* test, two‐way ANOVA and log‐rank test were performed to assess statistical significance between groups as indicated in the legends. *p*‐values <.05 were considered statistically significant.

## CONFLICT OF INTEREST

The authors declare that they have no conflict of interest.

## AUTHORS' CONTRIBUTION

JF, HZ, CCW, GHL, and LW designed the study; JF, JY, XW, GZ, TL, and XEW performed research; JF, JY, XW, and LW analyzed data; and JF, JY, CCW, GHL, and LW wrote the manuscript.

## References

[acel12765-bib-0001] Algire, C. , Moiseeva, O. , Deschenes‐Simard, X. , Amrein, L. , Petruccelli, L. , Birman, E. , … Pollak, M. N. (2012). Metformin reduces endogenous reactive oxygen species and associated DNA damage. Cancer Prevention Research, 5, 536–543. 10.1158/1940-6207.CAPR-11-0536 22262811

[acel12765-bib-0002] Bailey, C. J. , & Day, C. (2004). Metformin: Its botanical background. Practical Diabetes, 21, 115–117. 10.1002/(ISSN)1528-252X

[acel12765-bib-0003] Bannister, C. A. , Holden, S. E. , Jenkins‐Jones, S. , Morgan, C. L. , Halcox, J. P. , Schernthaner, G. , … Currie, C. J. (2014). Can people with type 2 diabetes live longer than those without? A comparison of mortality in people initiated with metformin or sulphonylurea monotherapy and matched, non‐diabetic controls. Diabetes, Obesity & Metabolism, 16, 1165–1173. 10.1111/dom.12354 25041462

[acel12765-bib-0004] Barzilai, N. , Crandall, J. P. , Kritchevsky, S. B. , & Espeland, M. A. (2016). Metformin as a tool to target aging. Cell Metabolism, 23, 1060–1065. 10.1016/j.cmet.2016.05.011 27304507 PMC5943638

[acel12765-bib-0005] Cabreiro, F. , Au, C. , Leung, K. Y. , Vergara‐Irigaray, N. , Cocheme, H. M. , Noori, T. , … Gems, D. (2013). Metformin retards aging in *C. elegans* by altering microbial folate and methionine metabolism. Cell, 153, 228–239. 10.1016/j.cell.2013.02.035 23540700 PMC3898468

[acel12765-bib-0006] Cullinan, S. B. , Zhang, D. , Hannink, M. , Arvisais, E. , Kaufman, R. J. , & Diehl, J. A. (2003). Nrf2 is a direct PERK substrate and effector of PERK‐dependent cell survival. Molecular and Cellular Biology, 23, 7198–7209. 10.1128/MCB.23.20.7198-7209.2003 14517290 PMC230321

[acel12765-bib-0007] Dahl, J. A. , & Collas, P. (2008). A rapid micro chromatin immunoprecipitation assay (microChIP). Nature Protocols, 3, 1032–1045. 10.1038/nprot.2008.68 18536650

[acel12765-bib-0008] De Haes, W. , Frooninckx, L. , Van Assche, R. , Smolders, A. , Depuydt, G. , Billen, J. , … Temmerman, L. (2014). Metformin promotes lifespan through mitohormesis via the peroxiredoxin PRDX‐2. Proceedings of the National Academy of Sciences of the United States of America, 111, E2501–E2509. 10.1073/pnas.1321776111 24889636 PMC4066537

[acel12765-bib-0009] Delaunay‐Moisan, A. , & Appenzeller‐Herzog, C. (2015). The antioxidant machinery of the endoplasmic reticulum: Protection and signaling. Free Radical Biology and Medicine, 83, 341–351. 10.1016/j.freeradbiomed.2015.02.019 25744411

[acel12765-bib-0010] Duan, S. L. , Yuan, G. H. , Liu, X. M. , Ren, R. T. , Li, J. Y. , Zhang, W. Z. , … Liu, G. H. (2015). PTEN deficiency reprogrammes human neural stem cells towards a glioblastoma stem cell‐like phenotype. Nature Communications, 6, 10068 10.1038/ncomms10068 PMC4686761 26632666

[acel12765-bib-0011] Finkel, T. (2011). Signal transduction by reactive oxygen species. Journal of Cell Biology, 194, 7–15. 10.1083/jcb.201102095 21746850 PMC3135394

[acel12765-bib-0012] Foretz, M. , Hebrard, S. , Leclerc, J. , Zarrinpashneh, E. , Soty, M. , Mithieux, G. , … Viollet, B. (2010). Metformin inhibits hepatic gluconeogenesis in mice independently of the LKB1/AMPK pathway via a decrease in hepatic energy state. The Journal of Clinical Investigation, 120, 2355–2369. 10.1172/JCI40671 20577053 PMC2898585

[acel12765-bib-0013] Gozzelino, R. , Jeney, V. , & Soares, M. P. (2010). Mechanisms of cell protection by heme oxygenase‐1. Annual Review of Pharmacology and Toxicology, 50, 323–354. 10.1146/annurev.pharmtox.010909.105600 20055707

[acel12765-bib-0014] Han, S. K. , Lee, D. , Lee, H. , Kim, D. , Son, H. G. , Yang, J. S. , … Kim, S. (2016). OASIS 2: Online application for survival analysis 2 with features for the analysis of maximal lifespan and healthspan in aging research. Oncotarget, 7, 56147–56152. 27528229 10.18632/oncotarget.11269 PMC5302902

[acel12765-bib-0015] He, L. , & Wondisford, F. E. (2015). Metformin action: Concentrations matter. Cell Metabolism, 21, 159–162. 10.1016/j.cmet.2015.01.003 25651170

[acel12765-bib-0016] Holden‐Dye, L. , & Walker, R. J. (2007) Anthelmintic drugs. WormBook, 1–13. 10.1895/wormbook.1.143.1 PMC4781348 17988075

[acel12765-bib-0017] Hourihan, J. M. , Moronetti Mazzeo, L. E. , Fernandez‐Cardenas, L. P. , & Blackwell, T. K. (2016). Cysteine sulfenylation directs IRE‐1 to activate the SKN‐1/Nrf2 antioxidant response. Molecular Cell, 63, 553–566. 10.1016/j.molcel.2016.07.019 27540856 PMC4996358

[acel12765-bib-0018] Kirstein, J. , Morito, D. , Kakihana, T. , Sugihara, M. , Minnen, A. , Hipp, M. S. , … Morimoto, R. I. (2015). Proteotoxic stress and ageing triggers the loss of redox homeostasis across cellular compartments. EMBO Journal, 34, 2334–2349. 10.15252/embj.201591711 26228940 PMC4570520

[acel12765-bib-0019] Konno, T. , Pinho Melo, E. , Lopes, C. , Mehmeti, I. , Lenzen, S. , Ron, D. , & Avezov, E. (2015). ERO1‐independent production of H2O2 within the endoplasmic reticulum fuels Prdx4‐mediated oxidative protein folding. Journal of Cell Biology, 211, 253–259. 10.1083/jcb.201506123 26504166 PMC4621842

[acel12765-bib-0020] Kubben, N. , Zhang, W. , Wang, L. , Voss, T. C. , Yang, J. , Qu, J. , … Misteli, T. (2016). Repression of the antioxidant NRF2 pathway in premature aging. Cell, 165, 1361–1374. 10.1016/j.cell.2016.05.017 27259148 PMC4893198

[acel12765-bib-0021] Li, C. , Shi, L. , Chen, D. , Ren, A. , Gao, T. , & Zhao, M. (2015). Functional analysis of the role of glutathione peroxidase (GPx) in the ROS signaling pathway, hyphal branching and the regulation of ganoderic acid biosynthesis in *Ganoderma lucidum* . Fungal Genetics and Biology, 82, 168–180. 10.1016/j.fgb.2015.07.008 26216672

[acel12765-bib-0022] Li, Y. , Zhang, W. , Chang, L. , Han, Y. , Sun, L. , Gong, X. , … Liu, G. H. (2016). Vitamin C alleviates aging defects in a stem cell model for Werner syndrome. Protein & Cell, 7, 478–488. 10.1007/s13238-016-0278-1 27271327 PMC4930768

[acel12765-bib-0023] Liu, G. H. , Suzuki, K. , Li, M. , Qu, J. , Montserrat, N. , Tarantino, C. , … Izpisua Belmonte, J. C. (2014). Modelling Fanconi anemia pathogenesis and therapeutics using integration‐free patient‐derived iPSCs. Nature Communications, 5, 4330. 10.1038/ncomms5330 PMC4291073 24999918

[acel12765-bib-0024] Longo, V. D. , Antebi, A. , Bartke, A. , Barzilai, N. , Brown‐Borg, H. M. , Caruso, C. , … Fontana, L. (2015). Interventions to slow aging in humans: Are we ready? Aging Cell, 14, 497–510. 10.1111/acel.12338 25902704 PMC4531065

[acel12765-bib-0025] Lopez‐Otin, C. , Blasco, M. A. , Partridge, L. , Serrano, M. , & Kroemer, G. (2013). The hallmarks of aging. Cell, 153, 1194–1217. 10.1016/j.cell.2013.05.039 23746838 PMC3836174

[acel12765-bib-0026] Ma, Q. (2013). Role of nrf2 in oxidative stress and toxicity. Annual Review of Pharmacology and Toxicology, 53, 401–426. 10.1146/annurev-pharmtox-011112-140320 PMC4680839 23294312

[acel12765-bib-0027] Martin‐Castillo, B. , Vazquez‐Martin, A. , Oliveras‐Ferraros, C. , & Menendez, J. A. (2010). Metformin and cancer: Doses, mechanisms and the dandelion and hormetic phenomena. Cell Cycle, 9, 1057–1064. 10.4161/cc.9.6.10994 20305377

[acel12765-bib-0028] Martin‐Montalvo, A. , Mercken, E. M. , Mitchell, S. J. , Palacios, H. H. , Mote, P. L. , Scheibye‐Knudsen, M. , … de Cabo, R. (2013). Metformin improves healthspan and lifespan in mice. Nature Communications, 4, 2192. 10.1038/ncomms3192 PMC3736576 23900241

[acel12765-bib-0029] Menendez, J. A. , Cufi, S. , Oliveras‐Ferraros, C. , Martin‐Castillo, B. , Joven, J. , Vellon, L. , & Vazquez‐Martin, A. (2011). Metformin and the ATM DNA damage response (DDR): Accelerating the onset of stress‐induced senescence to boost protection against cancer. Aging (Albany NY), 3, 1063–1077. 10.18632/aging.100407 22170748 PMC3249452

[acel12765-bib-0030] Nguyen, V. D. , Saaranen, M. J. , Karala, A. R. , Lappi, A. K. , Wang, L. , Raykhel, I. B. , … Ruddock, L. W. (2011). Two endoplasmic reticulum PDI peroxidases increase the efficiency of the use of peroxide during disulfide bond formation. Journal of Molecular Biology, 406, 503–515. 10.1016/j.jmb.2010.12.039 21215271

[acel12765-bib-0031] Noren Hooten, N. , Martin‐Montalvo, A. , Dluzen, D. F. , Zhang, Y. , Bernier, M. , Zonderman, A. B. , … Evans, M. K. (2016). Metformin‐mediated increase in DICER1 regulates microRNA expression and cellular senescence. Aging Cell, 15, 572–581. 10.1111/acel.12469 26990999 PMC4854919

[acel12765-bib-0032] Novelle, M. G. , Ali, A. , Dieguez, C. , Bernier, M. , & de Cabo, R. (2016). Metformin: A hopeful promise in aging research. Cold Spring Harbor Perspectives in Medicine, 6, a025932 10.1101/cshperspect.a025932 26931809 PMC4772077

[acel12765-bib-0033] Oh, J. , Lee, Y. D. , & Wagers, A. J. (2014). Stem cell aging: Mechanisms, regulators and therapeutic opportunities. Nature Medicine, 20, 870–880. 10.1038/nm.3651 PMC4160113 25100532

[acel12765-bib-0034] Onken, B. , & Driscoll, M. (2010). Metformin induces a dietary restriction‐like state and the oxidative stress response to extend *C. elegans* Healthspan via AMPK, LKB1, and SKN‐1. PLoS One, 5, e8758 10.1371/journal.pone.0008758 20090912 PMC2807458

[acel12765-bib-0035] Orrenius, S. , Gogvadze, V. , & Zhivotovsky, B. (2007). Mitochondrial oxidative stress: Implications for cell death. Annual Review of Pharmacology and Toxicology, 47, 143–183. 10.1146/annurev.pharmtox.47.120505.105122 17029566

[acel12765-bib-0036] Pan, H. , Guan, D. , Liu, X. , Li, J. , Wang, L. , Wu, J. , … Liu, G. H. (2016). SIRT6 safeguards human mesenchymal stem cells from oxidative stress by coactivating NRF2. Cell Research, 26, 190–205. 10.1038/cr.2016.4 26768768 PMC4746611

[acel12765-bib-0037] Peng, D. , Belkhiri, A. , Hu, T. , Chaturvedi, R. , Asim, M. , Wilson, K. T. , … El‐Rifai, W. (2012). Glutathione peroxidase 7 protects against oxidative DNA damage in oesophageal cells. Gut, 61, 1250–1260. 10.1136/gutjnl-2011-301078 22157330 PMC3419307

[acel12765-bib-0038] Pernicova, I. , & Korbonits, M. (2014). Metformin–mode of action and clinical implications for diabetes and cancer. Nature Reviews Endocrinology, 10, 143–156. 10.1038/nrendo.2013.256 24393785

[acel12765-bib-0039] Ramming, T. , Hansen, H. G. , Nagata, K. , Ellgaard, L. , & Appenzeller‐Herzog, C. (2014). GPx8 peroxidase prevents leakage of H2O2 from the endoplasmic reticulum. Free Radical Biology and Medicine, 70, 106–116. 10.1016/j.freeradbiomed.2014.01.018 24566470

[acel12765-bib-0040] Rhee, S. G. , Woo, H. A. , Kil, I. S. , & Bae, S. H. (2012). Peroxiredoxin functions as a peroxidase and a regulator and sensor of local peroxides. Journal of Biological Chemistry, 287, 4403–4410. 10.1074/jbc.R111.283432 22147704 PMC3281607

[acel12765-bib-0041] Smith, D. L. Jr , Elam, C. F. Jr , Mattison, J. A. , Lane, M. A. , Roth, G. S. , Ingram, D. K. , & Allison, D. B. (2010). Metformin supplementation and life span in Fischer‐344 rats. Journals of Gerontology Series A: Biological Sciences and Medical Sciences, 65, 468–474. 10.1093/gerona/glq033 PMC2854888 20304770

[acel12765-bib-0042] Suh, J. H. , Shenvi, S. V. , Dixon, B. M. , Liu, H. , Jaiswal, A. K. , Liu, R. M. , & Hagen, T. M. (2004). Decline in transcriptional activity of Nrf2 causes age‐related loss of glutathione synthesis, which is reversible with lipoic acid. Proceedings of the National Academy of Sciences of the United States of America, 101, 3381–3386. 10.1073/pnas.0400282101 14985508 PMC373470

[acel12765-bib-0043] Turpaev, K. T. (2002). Reactive oxygen species and regulation of gene expression. Biochemistry (Moscow), 67, 281–292. 10.1023/A:1014819832003 11970728

[acel12765-bib-0044] Wang, S. , Hu, B. Q. , Ding, Z. C. , Dang, Y. J. , Wu, J. , Li, D. , … Liu, G. H. (2018). ATF6 safeguards organelle homeostasis and cellular aging in human mesenchymal stem cells. Cell Discovery, 4, 2 10.1038/s41421-017-0003-0 29423270 PMC5798892

[acel12765-bib-0045] Wang, L. , Zhang, L. , Niu, Y. , Sitia, R. , & Wang, C. C. (2014). Glutathione peroxidase 7 utilizes hydrogen peroxide generated by Ero1alpha to promote oxidative protein folding. Antioxidants & Redox Signaling, 20, 545–556. 10.1089/ars.2013.5236 23919619 PMC3901321

[acel12765-bib-0046] Wei, P. C. , Hsieh, Y. H. , Su, M. I. , Jiang, X. , Hsu, P. H. , Lo, W. T. , … Lee, W. H. (2012). Loss of the oxidative stress sensor NPGPx compromises GRP78 chaperone activity and induces systemic disease. Molecular Cell, 48, 747–759. 10.1016/j.molcel.2012.10.007 23123197 PMC3582359

[acel12765-bib-0047] Wu, L. , Zhou, B. , Oshiro‐Rapley, N. , Li, M. , Paulo, J. A. , Webster, C. M. , … Soukas, A. A. (2016). An ancient, unified mechanism for metformin growth inhibition in *C. elegans* and cancer. Cell, 167(1705–1718), e1713. 10.1016/j.cell.2016.11.055 PMC5390486 27984722

[acel12765-bib-0048] Yang, J. , Li, J. , Suzuki, K. , Liu, X. , Wu, J. , Zhang, W. , … Liu, G. H. (2017). Genetic enhancement in cultured human adult stem cells conferred by a single nucleotide recoding. Cell Research, 27, 1178–1181. 10.1038/cr.2017.86 28685772 PMC5587854

[acel12765-bib-0049] Zhang, W. , Li, J. , Suzuki, K. , Qu, J. , Wang, P. , Zhou, J. , … Belmonte, J. C. (2015). Aging stem cells. A Werner syndrome stem cell model unveils heterochromatin alterations as a driver of human aging. Science, 348, 1160–1163. 10.1126/science.aaa1356 25931448 PMC4494668

[acel12765-bib-0050] Zhou, G. , Myers, R. , Li, Y. , Chen, Y. , Shen, X. , Fenyk‐Melody, J. , … Moller, D. E. (2001). Role of AMP‐activated protein kinase in mechanism of metformin action. The Journal of Clinical Investigation, 108, 1167–1174. 10.1172/JCI13505 11602624 PMC209533

[acel12765-bib-0051] Zito, E. , Melo, E. P. , Yang, Y. , Wahlander, A. , Neubert, T. A. , & Ron, D. (2010). Oxidative protein folding by an endoplasmic reticulum‐localized peroxiredoxin. Molecular Cell, 40, 787–797. 10.1016/j.molcel.2010.11.010 21145486 PMC3026605

