# Supplementary material for: Metformin alleviates human cellular aging by upregulating the endoplasmic reticulum glutathione peroxidase 7
Source: Aging Cell. 2018 Apr 16;17(4):e12765. doi: 10.1111/acel.12765 (PMC6052468; doi:10.1111/acel.12765)
Supplement: Supplementary file 1 [file ACEL-17-na-s001.pdf]

Supporting Information

Supporting Experimental Procedures

Flow cytometry analysis.

For cell apoptosis analysis, cells were collected freshly and stained with Annexin V-Alexa Fluor 488/PI (Life Technologies) for 15 min, and then apoptotic cells were quantified by FACS (BD FACS Calibur) with an excitation at 488 nm.

Western blotting.

Cells were lysed using lysis buffer (Millipore) with Protease Inhibitor Cocktail (Roche). Protein quantification was performed using a BCA Kit (Beyotime). Protein lysate was subjected to SDS-PAGE and subsequently electro transferred onto a polyvinylidene floride membrane (Millipore). Blots were developed by indicated antibodies and enhanced chemiluminescence (ECL) (Millipore), followed by using a ChemiScope mini chemiluminescence imaging system (Clinx Science). The antibodies used are listed as follows: anti-Nrf2 (Abcam, ab62352, 1: 1000), anti-AMPK (CST, 2532, 1: 1000), anti-*p*-AMPK (CST, 2535, 1: 1000), anti-PERK (CST, 5683, 1: 1000), goat anti-rabbit IgG (Sigma, A0545, 1: 10,000), goat anti-mouse IgG (Sigma, A4416, 1: 10,000).

Cell culture.

BJ-1 fibroblasts (from ATCC) were cultured in DMEM (Gibco, 11995-065) supplemented with 10% fetal bovine serum (Gibco, 10099-141), 0.1 mM non-essential amino acids (Gibco), 1% penicillin/streptomycin (Gibco).

Figure S1. High concentration of metformin accelerates the senescence of HDFs. (A) HDFs were treated with PBS (Ctrl) or different concentration of metformin (Met) as indicated for 24 h, and the apoptotic cells were determined by Annexin V/PI staining using FACS. (B) Cumulative population doubling (CPD) analysis of HDFs continuously cultured in the absence (Ctrl) or presence of 1 mM or 10 mM metformin, respectively.

Figure S2. Low concentration of metformin does not activate AMPK in HDFs. Western blotting analysis of AMPK and phosphorylated AMPK (*p*-AMPK) in HDFs by administration of indicated concentration of metformin for 24 h.

Figure S3. Metformin upregulates GPx7 and GPx7 retards the senescence of BJ-1 fibroblasts induced by *NRF2* depletion. (A) *Upper:* GPx7 in BJ-1 fibroblasts treated with indicated concentrations of metformin for 24 h. *Lower:* Statistical analysis of the expression of GPx7. Data were represented as mean ± SEM from 4 biological replicates. * $P < 0.05$, ** $P < 0.01$, via two tailed Student's *t* test. (B) CPD analysis of BJ-1 fibroblasts proliferation in the absence (Ctrl) or presence of 10 μM metformin. (C) *Left:* Senescence-associated-β-Galactosidase (SA-β-Gal) staining of BJ-1 fibroblasts at passage 12 (P12). Scale bar = 100 μm. *Right:* Statistical analysis of the percentages of SA-β-Gal-positive cells. Data were represented as mean ± SEM from 3 biological replicates, n > 200 cells per condition. ** $P < 0.01$ via two tailed Student's *t* test. (D) Western blotting to detect the efficiency of knockdown of NRF2 and overexpression of GPx7 by lentivirus as indicated in BJ-1 fibroblasts. (E, F) *Left:* SA-β-Gal staining (E) and KI67 expression (F) of BJ-1 fibroblasts at P7. Scale bar = 50 and 20 μm in (E) and (F), respectively. *Right:* Statistical analysis of the percentages of SA-β-Gal-positive cells and KI67-positive cells were illustrated. Data were represented as mean ± SEM from 3 biological replicates, n > 200 cells per condition. ** $P < 0.01$ via two-way ANOVA, Tukey's multiple comparisons test.

Figure S4. Knocking down of *GPX7* accelerates the senescence phenotype of HDFs. (A, B) *Left:* SA-β-Gal staining (A) and KI67 expression (B) of HDFs transduced with lentiviral shCtrl or sh*GPX7* at LP. Scale bar = 50 and 20 μm in (A) and (B), respectively. *Right:* Statistical analysis of the percentages of SA-β-Gal-positive cells and KI67-positive cells were illustrated. (C) CPD analysis of HDFs proliferation transduced with lentiviral GFP and GPx7. *Inset*: GPx7 expression in HDFs transduced with lentivirus. Data were represented as mean ± SEM from 3 biological replicates, n > 200 cells. * $P < 0.05$, ** $P < 0.01$ via two tailed Student's *t* test.

Figure S5. ChIP-qPCR analysis of the Nrf2 occupancy on GPX7 promoter. *Upper*: Localization of the ARE-containing sites (S1) and one non-specific site (S2) at the GPX7 promoter. *Lower*: ChIP-qPCR analysis was performed with IgG or anti-Nrf2 antibody in HDFs with or without 200 μM tBHQ treatment for 24 h. Enrichment values were normalized to input, and shown as the fold changes relative to IgG group. Data were represented as mean ± SEM from 3 technical replicates, ** $P < 0.01$, n.s., not significant, via two-way ANOVA, Tukey's multiple comparisons test.

Figure S6. A model illustrating generation of the endogenous HMSC-*NRF2*$^{AG/AG}$. A single nucleotide variation (A254G) of *NRF2* gene were introduced in the genome via homologous recombination-based gene editing. This variation results in a glutamic acid to glycine switch at amino acid 82 in the Nrf2 protein, which is supposed to release Nrf2 from its repressor KEAP1 and lead to Nrf2 stabilization and transcriptional activation of its target genes.

Figure S7. Identification of the ortholog of human GPx7 in *C. elegans*. (A) Protein sequence alignments between human GPx7, *C. elegans* GPX-6 and GPX-7 by T coffee (Version_11.00.8cbe486). (B) Signal peptide prediction of GPX-6 and GPX-7 of *C. elegans* by SingalP-4.1. (C) *Upper: mcherry* was translationally fused to the full-length genomic DNA of *gpx-7*. The expression was driven by the *y37a1b.5* promoter. *Lower:* Transgenic worms simultaneously expressed GPX-7 [*Py37a1b.5::gpx-7::mcherry*] (red channel) and TRAM-1 [*Py37a1b.5::tram-1::gfp*] (green channel). Scale bar = 5 μm.

Figure S8. Role of *C. elegans* GPX-6 against oxidative stress. (A) Images and quantification of the worms expressing GPX-6::GFP subjected to DMSO (Ctrl) or 500 μM tBHQ for 48 h post L4 larval stage. Scale bars = 100 μm. Data were represented as mean ± SEM, n > 15 worms per condition. ** $P < 0.01$ via two tailed Student's *t* test. (B) The expression of GPX-6::GFP on day 1, day 5 and day 10. Scale bars = 100 μm. Data were represented as mean ± SEM, n > 20 worms per condition. ** $P < 0.01$ via two tailed Student's *t* test. (C) The efficiency of knockdown of *gpx-6* by RNAi feeding from L1 to young adult. Scale bars = 100 μm. Data were represented as mean ± SEM, n > 20 worms per condition. ** $P < 0.01$ via two tailed Student's *t* test. (D) Sensitivity to paraquat (PQ). Worms on Day 3 were transferred to M9 buffer containing 20 mM paraquat and monitored as indicated for survival. Data were represented as mean ± SEM from 3 technical replicates, n > 50 worms per condition. Results are representative of 2 independent experiments. ** $P < 0.01$ via two tailed Student's *t* test.

Figure S9 Low dose metformin activates Nrf2 in a PERK-independent manner. Protein expression of Nrf2, phosphorylation of PERK and total PERK in HDFs at P30 treated without or with 100 μM metformin for 24 h or with 1 μM thapsigargin (Tg) for 4h, respectively.

Figure S1

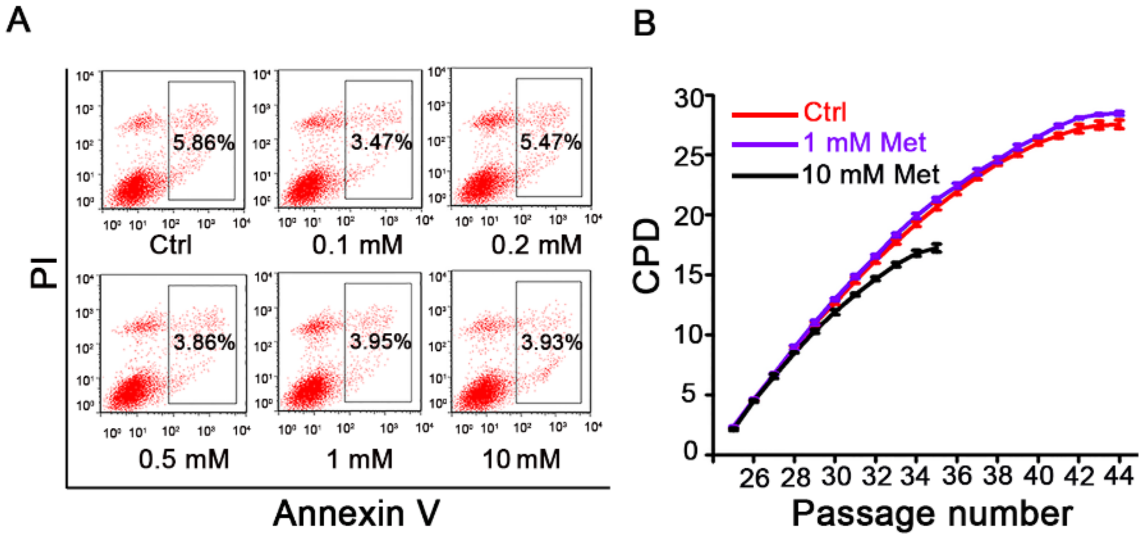
A
5.86%
Ctrl
3.47%
0.1 mM
5.47%
0.2 mM
3.86%
0.5 mM
3.95%
1 mM
3.93%
10 mM
PI
Annexin V
B
Ctrl
1 mM Met
10 mM Met
CPD
Passage number

Figure S2

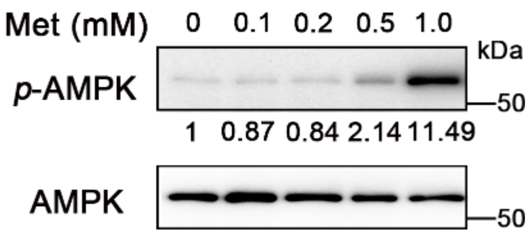

Figure S3

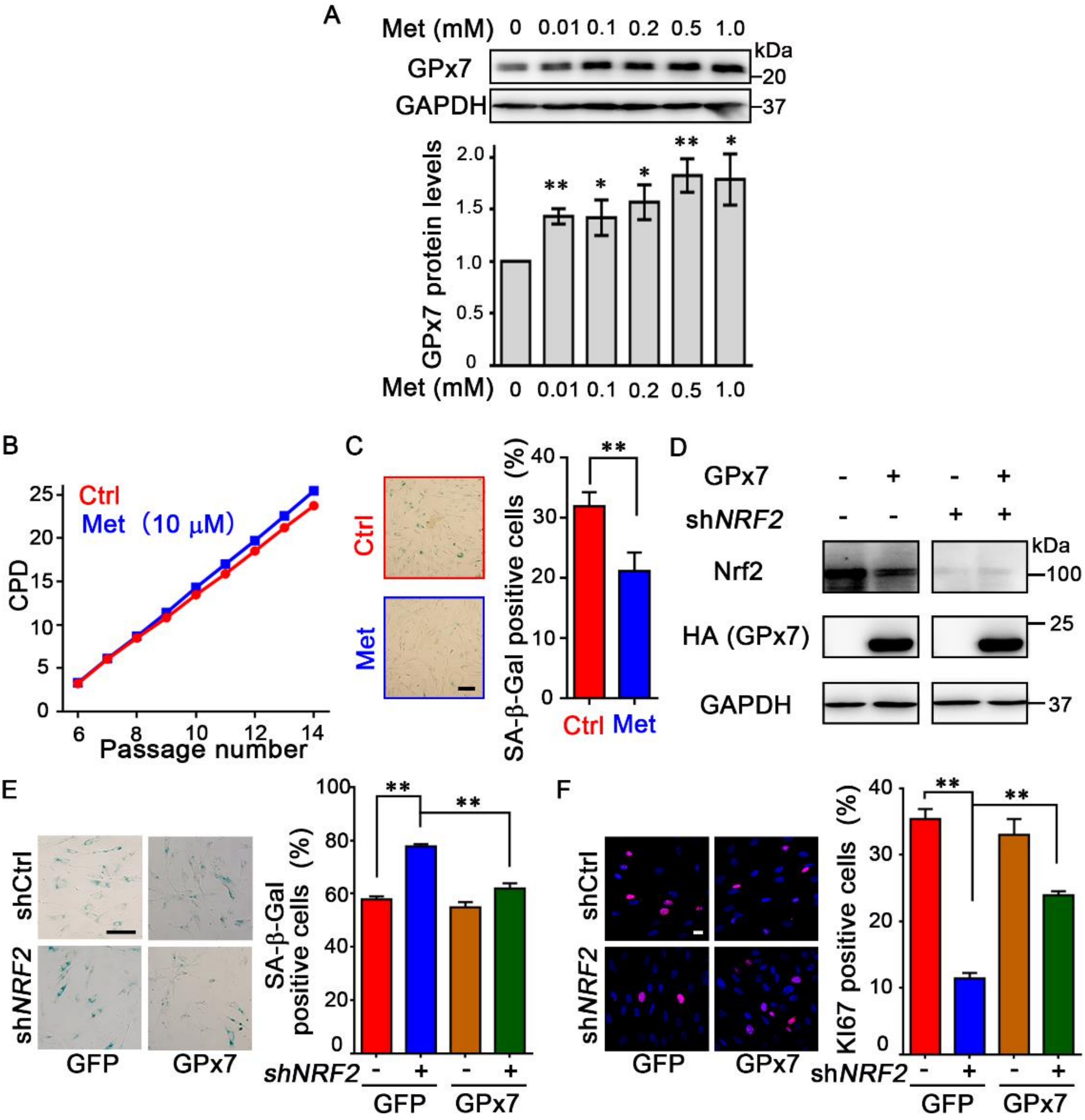

A
Met (mM) 0 0.01 0.1 0.2 0.5 1.0
GPx7
GAPDH
kDa
20
37
GPx7 protein levels
2.0
1.5
1.0
0.5
0
**
*
*
**
*
Met (mM) 0 0.01 0.1 0.2 0.5 1.0
B
Ctrl
Met (10 μM)
CPD
25
20
15
10
5
0
6 8 10 12 14
Passage number
C
Ctrl
Met
SA-β-Gal positive cells (%)
40
30
20
10
0
**
Ctrl Met
D
GPx7 - + - +
shNRF2 - - + +
Nrf2
HA (GPx7)
GAPDH
kDa
100
25
37
E
shCtrl
shNRF2
GFP
GPx7
SA-β-Gal positive cells (%)
100
80
60
40
20
0
**
**
shNRF2 - + - +
GFP
GPx7
F
shCtrl
shNRF2
GFP
GPx7
KI67 positive cells (%)
40
30
20
10
0
**
**
shNRF2 - + - +
GFP
GPx7

Figure S4

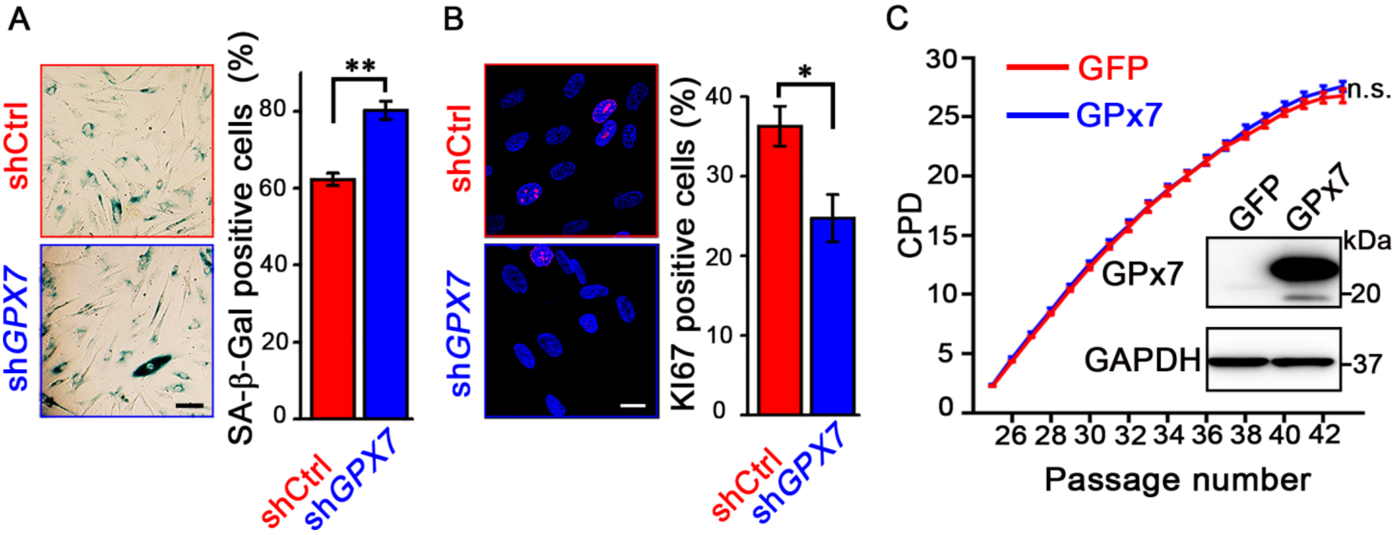
A
shCtrl
shGPX7
SA-β-Gal positive cells (%)
**
0
20
40
60
80
shCtrl
shGPX7
B
shCtrl
shGPX7
KI67 positive cells (%)
*
0
10
20
30
40
shCtrl
shGPX7
C
GFP
GPx7
CPD
0
5
10
15
20
25
30
n.s.
26 28 30 32 34 36 38 40 42
Passage number
GFP
GPx7
kDa
GPx7
-20
GAPDH
-37

Figure S5

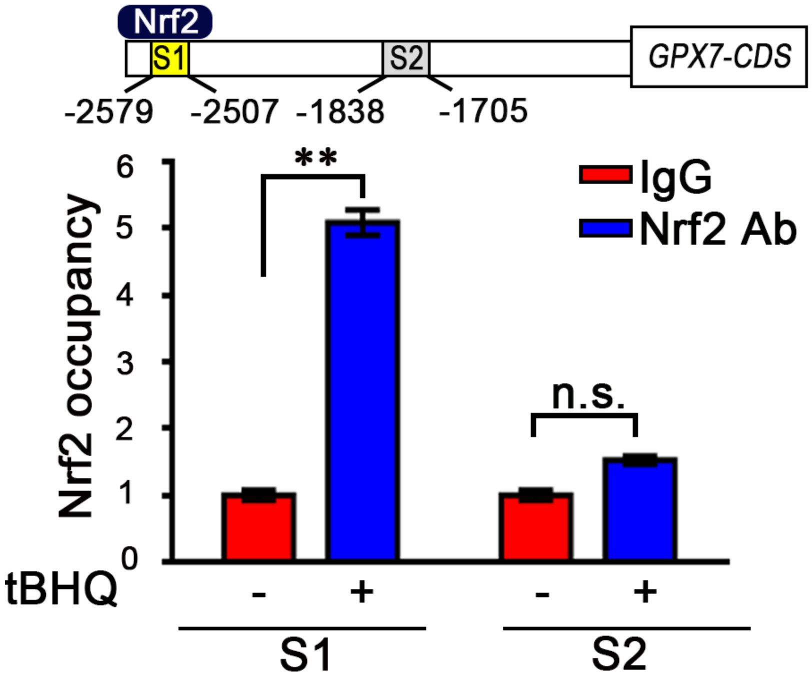

Nrf2
S1
S2
GPX7-CDS
-2579
-2507
-1838
-1705
Nrf2 occupancy
6
5
4
3
2
1
0
**
n.s.
IgG
Nrf2 Ab
tBHQ
-
+
-
+
S1
S2

Figure S6

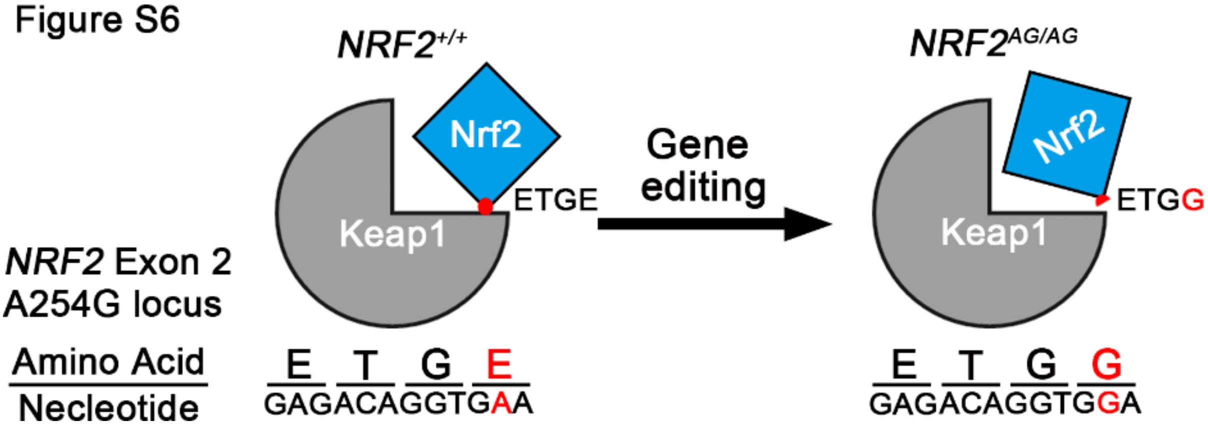
NRF2+/+
Nrf2
Keap1
ETGE
Gene editing
NRF2AG/AG
Nrf2
Keap1
ETGG
NRF2 Exon 2
A254G locus
Amino Acid
Necleotide
E T G E
GAGACAGGTGAA
E T G G
GAGACAGGTGGA

Figure S7

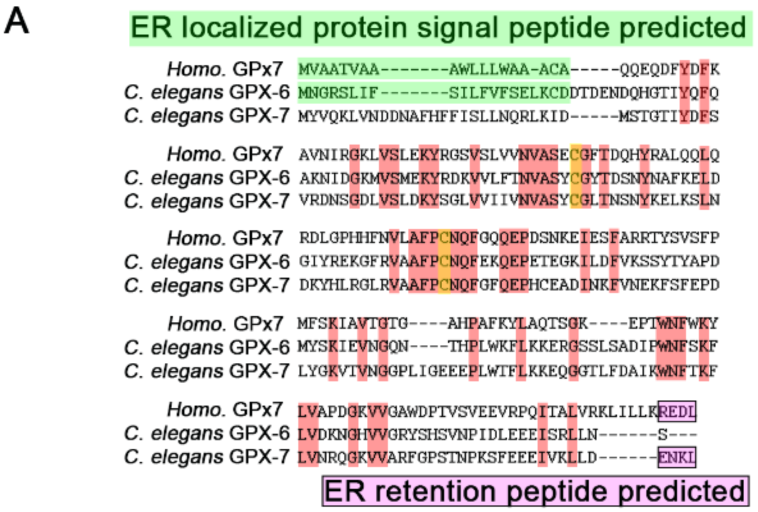
A
ER localized protein signal peptide predicted
Homo. GPx7 MVAATVAA-------AWLLLWAA-ACA-----QQEQDFYDFK
C. elegans GPX-6 MNGRSLIF-------SILFVFSELKCDDTDENDQHGTIYQFQ
C. elegans GPX-7 MYVQKLVNDDNAFHFFISLLNQRLKID-----MSTGTIYDFS
Homo. GPx7 AVNIRGKLVSLEKYRGSVSLVVNVASECGFTDQHYRALQQLQ
C. elegans GPX-6 AKNIDGKMVSMEKYRDKVVLFTNVASYCGYTDSNYNAFKELD
C. elegans GPX-7 VRDNSGDLVSLDKYSGLVVIIVNVASYCGLTNSNYKELKSLN
Homo. GPx7 RDLGPHHFNVLAFPCNQFGQQEPDSNKEIESFARRTYSVSFP
C. elegans GPX-6 GIYREKGFRVAAFPCNQFEKQEPETEGKILDFVKSSYTYAPD
C. elegans GPX-7 DKYHLRGLRVAAFPCNQFGFQEPHCEADINKFVNEKFSFEPD
Homo. GPx7 MFSKIAVTGTG----AHPAFKYLAQTSGK----EPTWNFWKY
C. elegans GPX-6 MYSKIEVNGQN----THPLWKFLKKERGSSLSADIPWNFSKF
C. elegans GPX-7 LYGKVTVNGGPLIGEEEPLWTFLKKEQGGTLFDAIKWNFTKF
Homo. GPx7 LVAPDGKVVGAWDPTVSVEEVRPQITALVRKLILLKREDL
C. elegans GPX-6 LVDKNGHVVGRYSHSVNPIDLEEEISRLLN------S---
C. elegans GPX-7 LVNRQGKVVARFGPSTNPKSFEEEIVKLLD------ENKI
ER retention peptide predicted

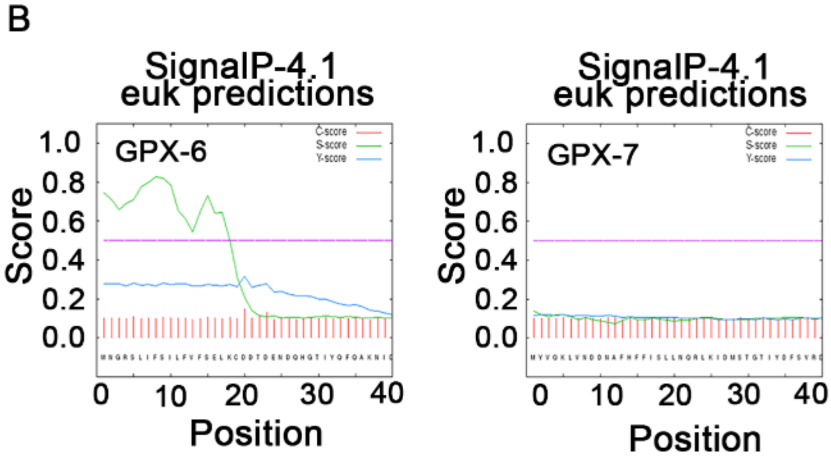
B
SignalP-4.1
euk predictions
GPX-6
GPX-7
C-score
S-score
Y-score
Score
1.0
0.8
0.6
0.4
0.2
0.0
0
10
20
30
40
Position

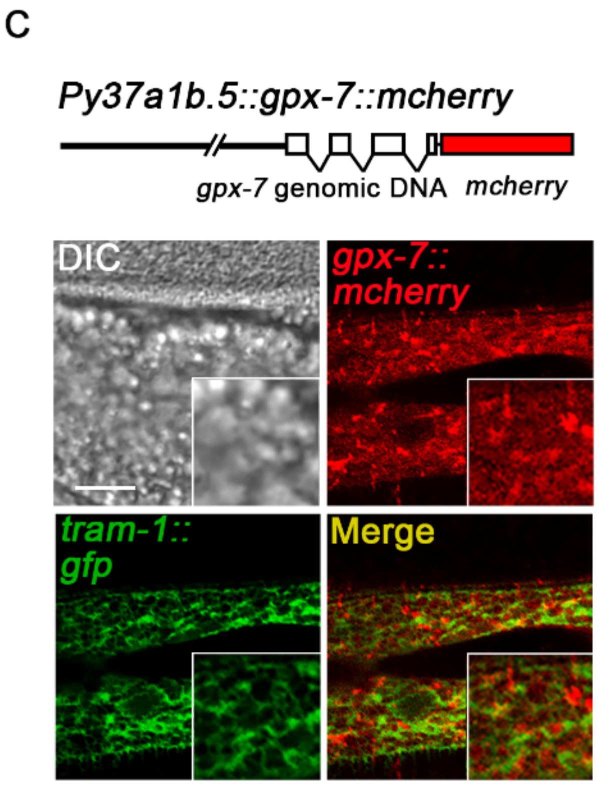
C
Py37a1b.5::gpx-7::mcherry
gpx-7 genomic DNA
mcherry
DIC
gpx-7::
mcherry
tram-1::
gfp
Merge

Figure S8

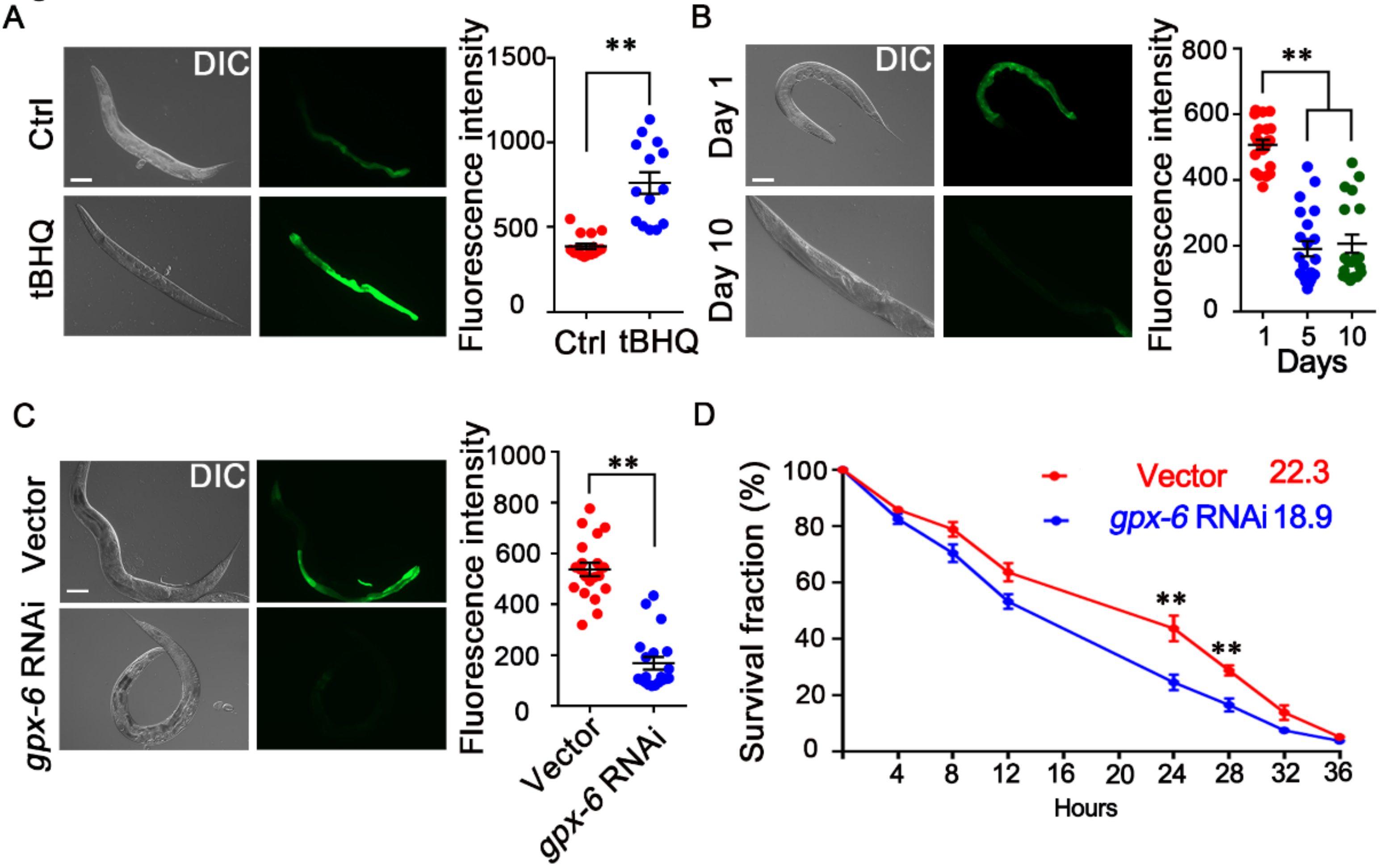
A
DIC
Ctrl
tBHQ
Fluorescence intensity
1500
1000
500
0
**
Ctrl
tBHQ
B
DIC
Day 1
Day 10
Fluorescence intensity
800
600
400
200
0
**
1
5
10
Days
C
DIC
Vector
gpx-6 RNAi
Fluorescence intensity
1000
800
600
400
200
0
**
Vector
gpx-6 RNAi
D
Survival fraction (%)
100
80
60
40
20
0
Vector 22.3
gpx-6 RNAi 18.9
**
**
4
8
12
16
20
24
28
32
36
Hours

Figure S9

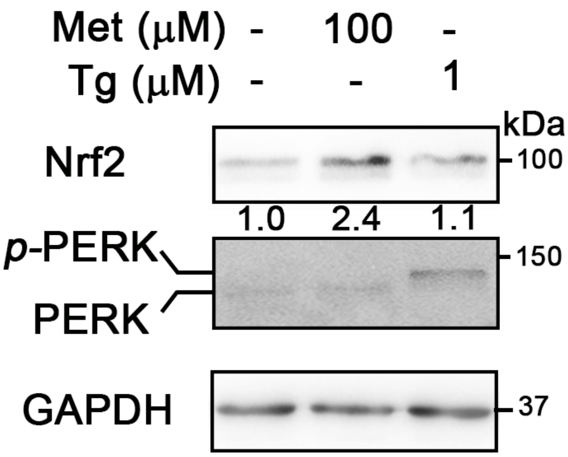

Table S1. Results of *C. elegans* lifespan experiments, related to Fig. 6E

No.1	Conditions	Numbers	Mean lifespan	SEM	Effect of metformin	*P* value (Log-Rank Test)
Ⅰ	Vector + Ctrl	84	16.1	0.6	Ⅰ vs Ⅱ, 16.1%	Ⅰ vs Ⅱ, 0.0012
Ⅱ	Vector + Met	83	18.7	0.7		Ⅰ vs Ⅲ, 0.0022
Ⅲ	*gpx-6* RNAi + Ctrl	87	13.8	0.5	Ⅲ vs Ⅳ, 5.8%	Ⅲ vs Ⅳ, 0.2455
Ⅳ	*gpx-6* RNAi + Met	82	14.6	0.6		

No.2	Conditions	Numbers	Mean lifespan	SEM	Effect of metformin	*P* value (Log-Rank Test)
Ⅰ	Vector + Ctrl	87	16.7	0.6	Ⅰ vs Ⅱ, 18.0%	Ⅰ vs Ⅱ, 0.0018
Ⅱ	Vector + Met	90	19.7	0.6		Ⅰ vs Ⅲ, 0.0005
Ⅲ	*gpx-6* RNAi + Ctrl	93	13.9	0.5	Ⅲ vs Ⅳ, 9.4%	Ⅲ vs Ⅳ, 0.1186
Ⅳ	*gpx-6* RNAi + Met	88	15.2	0.6		

No.3	Conditions	Numbers	Mean lifespan	SEM	Effect of metformin	*P* value (Log-Rank Test)
Ⅰ	Vector + Ctrl	91	17.1	0.6	Ⅰ *vs* Ⅱ, 15.8%	Ⅰ *vs* Ⅱ, 0.0022
Ⅱ	Vector + Met	87	19.8	0.7		Ⅰ *vs* Ⅲ,

						0.0018
Ⅲ	*gpx-6* RNAi + Ctrl	93	14.4	0.5	Ⅲ *vs* Ⅳ, 7.6%	Ⅲ *vs* Ⅳ, 0.2108
Ⅳ	*gpx-6* RNAi + Met	87	15.5	0.6		

**No.4**	**Conditions**	**Numbers**	**Mean lifespan**	**SEM**	**Effect of metformin**	***P* value (Log-Rank Test)**
Ⅰ	Vector + Ctrl	88	17.5	0.6	Ⅰ *vs* Ⅱ, 13.1%	Ⅰ *vs* Ⅱ, 0.0091
Ⅱ	Vector + Met	89	19.8	0.6		Ⅰ *vs* Ⅲ, 0.0027
Ⅲ	*gpx-6* RNAi + Ctrl	83	15	0.5	Ⅲ *vs* Ⅳ, 8.7%	Ⅲ *vs* Ⅳ, 0.1308
Ⅳ	*gpx-6* RNAi + Met	88	16.3	0.6		

The total numbers of tested worms, the mean lifespan ± SEM and the effect of metformin ((Met-Ctrl) / Ctrl × 100%) were listed.

Table S2. RT-qPCR primers used in Fig. 2A

**Gene name**	**Forward primer (5'--3')**	**Reverse primer (5'--3')**
GPX1	CAGTCGGTGTATGCCTTCTCG	GAGGGACGCCACATTCTCG
GPX3	AGAGCCGGGGACAAGAGAA	ATTTGCCAGCATACTGCTTGA
GPX4	GAGGCAAGACCGAAGTAAACTAC	CCGAACTGGTTACACGGGAA
GPX7	GACCAGCACTACCGAGCCCT	CCCCTACCACCTTTCCATCT
GPX8	CCGCCCAAGCAAGGAAGTAG	TCTAACCAGAGCTGCTATGTCAG
PRDX1	CCACGGAGATCATTGCTTTCA	AGGTGTATTGACCCATGCTAGAT
PRDX4	CTGTTGATTCACAGTTTACCCATTT	ATTATTGTTTCACTACCAGGTTTCC
SOD1	GGTGGGCCAAAGGATGAAGAG	CCACAAGCCAAACGACTTCC
SOD2	GGAAGCCATCAAACGTGACTT	CCCGTTCCTTATTGAAACCAAGC
CAT	TGGAGCTGGTAACCCAGTAGG	CCTTTGCCTTGGAGTATTTGGTA
HO1	AAGACTGCGTTCCTGCTCAAC	AAAGCCCTACAGCAACTGTCG
GAPDH	GGAGCGAGATCCCTCCAAAAT	GGCTGTTGTCATACTTCTCATGG

Table S3. The primers used for the construction of *GPX7* promoter regions in Fig. 3E.

Name	Sequence (5'--3')
GPX7-F1	GGGGTACCCAGCCTAAGGCCGCTGCACTAATGAGAAGC
GPX7-F2	GGGGTACCCATCTTAGCTCATCTGATGGGCTACTCTAG
GPX7-F3	GGGGTACCGATCTGCCTGCCTAGGCCTCCCAAAGTGCT
GPX7-F4	GGGGTACCACTCCAACATCTAGACCACATCTGCTCTTG
GPX7-F5	GGGGTACCACTCAAGGCCCTTCTCTATCCTGATGACCC
GPX7-F6	GGGGTACCATCACAGGCTTAAGGCTCCCAAAAGAGATG
GPX7-R	CCGCTCGAGGGCTTGTTCCGGAGGTGGCGGCGTCGCGAG

Table S4. The primers designed for ChIP-qPCR in Fig. 3F and Fig. S5.

**Name**	**Sequence (5'--3')**
***GPX7*-S1-F**	ACCTTTGCTTGCCACACTCT
***GPX7*-S1-R**	GATTGGAAGGGTCATCAGGA
***GPX7*-S2-F**	CCAGGATGGTCTCGTTCTC
***GPX7*-S2-R**	GGCAACCTAGCAAGAACTGTC

Primers *GPX7*-S1-F and *GPX7*-S1-R are for amplification of the -2579 ~ -2507 region, primers *GPX7*-S2-F and *GPX7*-S2-R are for amplification of the -1838 ~ -1705 region.

Table S5. The sequence synthesized for EMSA in Fig. 3G

Name	Sequence (5'--3')	5'-modification
***GPX7*-ARE-F**	CTAGAGAGTGACTTGGCACTCAA	Biotin
***GPX7*-ARE-R**	TTGAGTGCCAAGTCACTCTCTAG	Biotin
***HO1*-ARE-F**	ACTGAGGGTGACTCAGCAAAATC	
***HO1*-ARE-R**	GATTTTGCTGAGTCACCCTCAGT	
***mHO1*-ARE -F**	ACTGAGGGTGACTCAATAAAATC	
***mHO1*-ARE -R**	GATTT*TA*TTGAGTCACCCTCAGT	
